# Suppressing DUSP16 overexpression induced by ELK1 promotes neural progenitor cell differentiation in mouse models of Alzheimer's disease

**DOI:** 10.1111/acel.14372

**Published:** 2024-10-21

**Authors:** Huimin Zhao, Yao Mu, Anqi Liang, Jie Wei, Sixian Lai, Xin Li, Peipei Chen, Hao Li, Hua He, Xiaoquan Liu, Haochen Liu

**Affiliations:** ^1^ Center of Drug Metabolism and Pharmacokinetics China Pharmaceutical University Nanjing China; ^2^ Acupuncture and Moxibustion Department Jiangsu Provincial Second Chinese Medicine Hospital/the Second Affiliated Hospital of Nanjing University of Chinese Medicine Nanjing China

**Keywords:** Alzheimer's disease, cognitive function, DUSP16, hippocampal neurogenesis, synaptic transmission

## Abstract

Emerged evidence indicated that stimulating hippocampal neurogenesis is a potential strategy for restoring cognition in AD. Mitogen‐activated protein kinases (MAPKs) play an essential role in neurogenesis. Meanwhile, the enzymatic power of the phosphatases is much greater than that of kinases. Dual‐specificity phosphatase 16 (DUSP16), known to as a phosphatase negatively regulate MAPKs, may be implicated in neural differentiation. Nevertheless, the effect of DUSP16 on cognitive disorders by stimulating neural progenitor cell (NPC) differentiation in AD mice remains unclear. Our study demonstrates an association between DUSP16 SNPs and clinical progression in individuals with mild cognitive impairment (MCI). Besides, increased DUSP16 expression was detected in both 3xTg and SAMP8 mouse models of AD, accompanied by NPC neural differentiation impairments. By silencing DUSP16, the induction of neural differentiation, synaptic transmission, and cognitive benefits were observed in both AD mice. Furthermore, DUSP16 was involved in the process of NPC differentiation through regulating c‐Jun N‐terminal kinase (JNK) phosphorylation and SOX2 expression. Moreover, ETS transcription factor (ELK1) was involved in the DUSP16 transcription, which resulted in the upregulation of DUSP16 at the state of AD. Our data uncovers a potential regulatory role for DUSP16 in adult hippocampal neurogenesis (AHN) and provides a possibility to find a novel strategy for AD intervention.

## Graphical abstract

Elevated DUSP16 was observed in AD model mice, accompanied by deficits in hippocampal neurogenesis, synaptic transmission, and cognitive function. Silencing DUSP16 promoted the neural differentiation of NPCs, resulting in an increased generation of new neurons, improved synaptic transmission, and better cognitive performance.

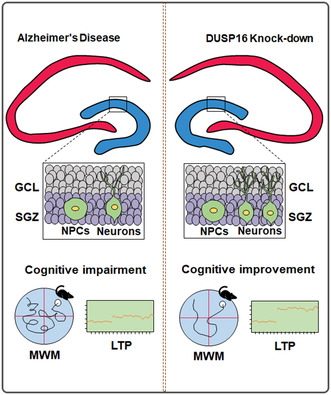

**Abbreviations**
AAV: adeno‐associated virus
ACSF: artificial cerebrospinal fluid
AD: Alzheimer's disease
AHN: adult hippocampal neurogenesis
BDNF: brain‐derived neurotrophic factor
CBP: CREB binding protein
DUSP16: dual‐specificity phosphatase 16
DUSPs: dual‐specificity phosphatases
ELK1: ETS transcription factor
fAD: familial AD
FGF: fibroblast growth factor
HFS: high‐frequency stimulation
I/O curve: input and output relationship (I/O) curve
IF: immunofluorescence
IP: immunoprecipitation
JNK: c‐Jun N‐terminal kinase
LTP: long‐term potentiation
MAPKs: mitogen‐activated protein kinases
MCI: mild cognitive impairment
MRI: magnetic resonance imaging
MWM: Morris water maze
NMDAR: N‐methyl D‐aspartate receptor
NPCs: neural progenitor cells
PET: positron emission tomography
PPF: paired‐pulse facilitation
PPR: paired‐pulse ratio
RMS: rostral migratory stream
sAD: sporadic AD
SGZ: subgranular zone
SNPs: single nucleotide polymorphisms
SOX2: SRY‐box transcription factor 2
SVZ: subventricular zone

## 1 INTRODUCTION

Alzheimer's disease (AD) is an age‐related neurodegenerative disease characterized by progressive memory loss and cognitive impairments (Scheltens et al., 2021). A major unmet challenge in current therapeutic interventions for AD is the limited effectiveness in improving cognitive function. There is strong evidence that restored neurogenesis could reverse the cognitive decline associated with AD, suggesting potential therapeutic relevance (Anacker & Hen, 2017).

Several lines of research have focused on engrafted regeneration and endogenous regeneration to relief defective neurogenesis (Sahay et al., 2011; Salwa & Kumar, 2021). Engrafted regeneration entails transplanting exogenous stem cells into the brain to restore degenerated neurons. Growing evidence has illuminated the potential of engrafted regeneration as a promising treatment strategy for AD, contributing to the restoration of cognition (Kang et al., 2016; Zhang, Jiang, et al., 2020). Nevertheless, the emerging problems such as immunological rejection, the targeted transplant area, and the low rate of neuronal differentiation, need to be settled before extending to AD clinical applications (Salwa & Kumar, 2021; Wang et al., 2018). Given these challenges, some researchers turn their eyes on endogenous regeneration. Despite the limited capacity for endogenous regeneration, more and more evidence have shown that restoring the impaired regenerative processes could appear as an effective strategy to prevent the onset and antagonize the progression of AD (Salta et al., 2023). Endogenous regeneration promotes the proliferation and differentiation of NPCs that reside in the hippocampus, partially compensating for damaged neurons (Zhao et al., 2008). Multiple lines of research indicate that hippocampal neurogenesis could be boosted through exercise, knockdown/overexpression of related genes, and pharmacological method, which attained the goal of ameliorating cognition in AD mouse models (Choi et al., 2018; Tiwari et al., 2014; Walgrave et al., 021). However, the molecular mechanisms involved in endogenous regeneration under AD conditions remain to be elucidated.

Since endogenous regeneration originates from hippocampal neurogenesis (Li & Clevers, 2010), the extracellular and intracellular factors which play essential roles in hippocampal neurogenesis are pivotal for endogenous regeneration. Extracellular factors encompass growth factors and neurotrophic factors, such as the brain‐derived neurotrophic factor (BDNF) and fibroblast growth factor (FGF); intracellular factors involve intracellular signaling pathways, such as the Notch, Wnt, BMP, and MAPK pathways (Denoth‐Lippuner & Jessberger, 2021). Numerous studies have revealed MAPK signaling could be activated by both extracellular and intracellular factors (Kouhara et al., 1997; Sapkota et al., 2007; Tursch et al., 2022). Therefore, MAPK pathway could serve as a hub of neurogenesis, enhanced by both extracellular factors and intracellular factors, indicating that MAPK pathway is essential for neurogenesis and neurite growth. For instance, hippocampal neurogenesis restoration and MAPK activity upregulation in AD mice were observed after physical exercise (Sun et al., 2018). Previous research has aimed to promote hippocampal neurogenesis by targeting the MAPK phosphorylation process, which could be activated by kinase regulation. While phosphatases inactivate the MAPK pathway through dephosphorylation (Arthur & Ley, 2013). Owing to the fact that the enzymatic power of a phosphatase is 100–1000 times more potent than that of a kinase (Reth, 2002). Thus, it is plausible that phosphatase expression and activity weigh more in constrained signal transduction compared to kinase activation (Jeffrey et al., 2007). Phosphatases that deactivate MAPKs encompass tyrosine phosphatases, serine/threonine phosphatases, and dual‐specificity phosphatases (DUSPs), which target phosphotyrosine and threonine residues in MAPK (Keyse, 1998). Recent studies have revealed the alterations of DUSPs in the hippocampal tissues from postmortem AD patients, suggesting the involvement of DUSPs in the pathophysiology of AD (Du et al., 2019; Jung et al., 2016). DUSPs appear to have more dynamic regulation on MAPK, we wonder whether regulating DUSPs has effectiveness on promoting hippocampal neurogenesis. Additionally, a transcriptomic study has identified an upregulation of DUSP16, a negative regulator of NPC proliferation and differentiation, in AD post‐mortem brain tissue (Miyashita et al., 2014; Zega et al., 2017). However, it remains uncertain whether the dephosphorylation function of DUSP16 is crucial for ameliorating cognitive disorders by affecting NPC differentiation in AD mice.

The correlation between DUSP16 SNPs and the risk of MCI progressing to AD risk was analyzed using ADNI clinical GWS data. Subsequently, transgenic mouse AD model 3xTg and non‐transgenic mouse AD model SAMP8 were used to investigate the potential improvements in hippocampal neurogenesis, synaptic transmission, and cognitive deficits through DUSP16 knockdown. Furthermore, the transcriptional regulatory mechanism of DUSP16 was also investigated. Our results provided a novel insight into the enhancement of NPC differentiation, which promises to address the unmet needs of patients with AD in terms of improved cognition.

## 2 RESULTS

### 2.1 DUSP16 specific SNPs are related to MCI‐to‐AD conversion risk

**FIGURE 1:**
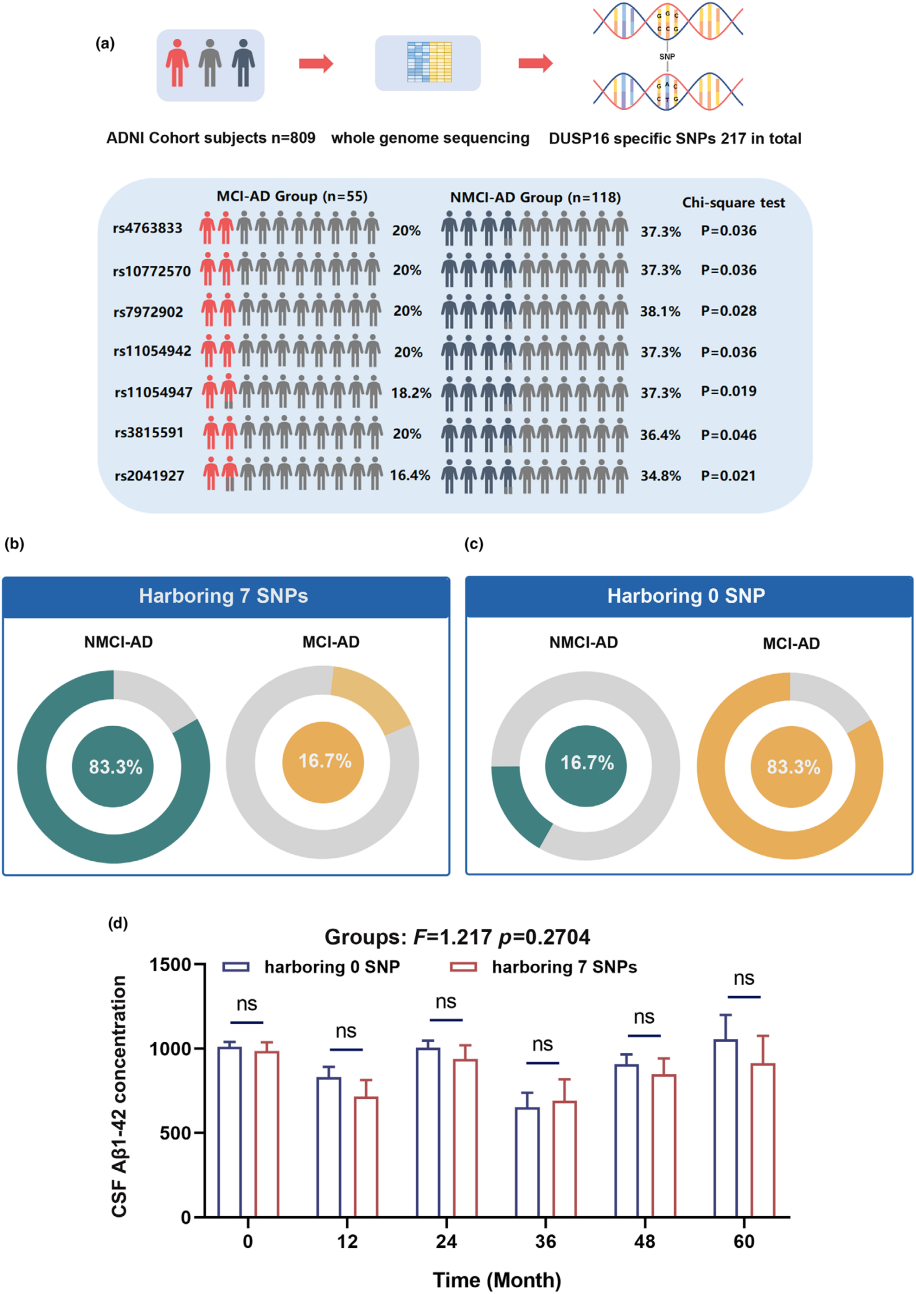
DUSP16 specific SNPs identify patients at risk for MCI‐to‐AD conversion. (a) The distribution of seven DUSP16 SNPs between the MCI‐AD subgroup (*n* = 55) and the NMCI‐AD subgroup (*n* = 118) group. (b) The subjects who harbor seven DUSP16 SNPs (a) attenuated rate of conversion from MCI to AD dementia. (c) The subjects who harbor exclusive seven DUSP16 SNPs (a) aggravated rate of conversion from MCI to AD dementia. (d) The CSF Aβ_1‐42_ concentration was compared at baseline and at 12, 24, 36, 60 months between the harboring 0 SNP cluster and the harboring 7 SNPs cluster. Chi‐square test was used in (a–c); Two‐way ANOVA was used in (d). Data are presented as mean ± SEM.

To explore the association between DUSP16 and AD risk, we analyzed a cohort with whole genome sequencing data from the ADNI database. The selected cohort contained 818 subjects, while 9 subjects were excluded due to failure in quality control. The 217 DUSP16 SNPs have been identified in this cohort. Initially, we compared the frequencies of these SNPs between the MCI‐to‐AD conversion group (patients who converted into AD within 18 months after they were diagnosed as MCI, *n* = 55) and the NMCI‐AD group (patients who did not convert into AD within 18 months after they were diagnosed as MCI, *n* = 118). 7 SNPs were discovered with significantly higher frequencies (Figure 1a; *p* < 0.05) in the NMCI‐AD subgroup compared to the MCI‐AD group. Given the presence of these 7 SNPs in many subjects, the two subgroups were further structured in two clusters: one containing 7 SNPs and the other containing none. Then the MCI‐to‐AD conversion risk of the two clusters was compared by chi‐square test (Table S2). The findings indicated that the patients who harbor 7 SNPs exhibited a markedly lower rate of MCI‐to‐AD conversion (Figure 1b,c; *p* < 0.05). Furthermore, the CSF Aβ_1‐42_ data of six time points were extracted within the ADNI database, excluding other points due to small sample sizes in two clusters (*n* < 5). No difference in CSF Aβ_1‐42_ concentration was found between the harboring 0 SNP cluster and the harboring 7 SNPs cluster at any time point (Figure 1d; *p* > 0.05). Collectively, our results demonstrate that the MCI‐to‐AD conversion proportion varies based on the presence of the 7 SNPs, suggesting that the DUSP16‐specific SNPs are associated with MCI‐to‐AD conversion risk.

### 2.2 Impaired neural differentiation of NPCs accompanied by elevated DUSP16 in AD

**FIGURE 2:**
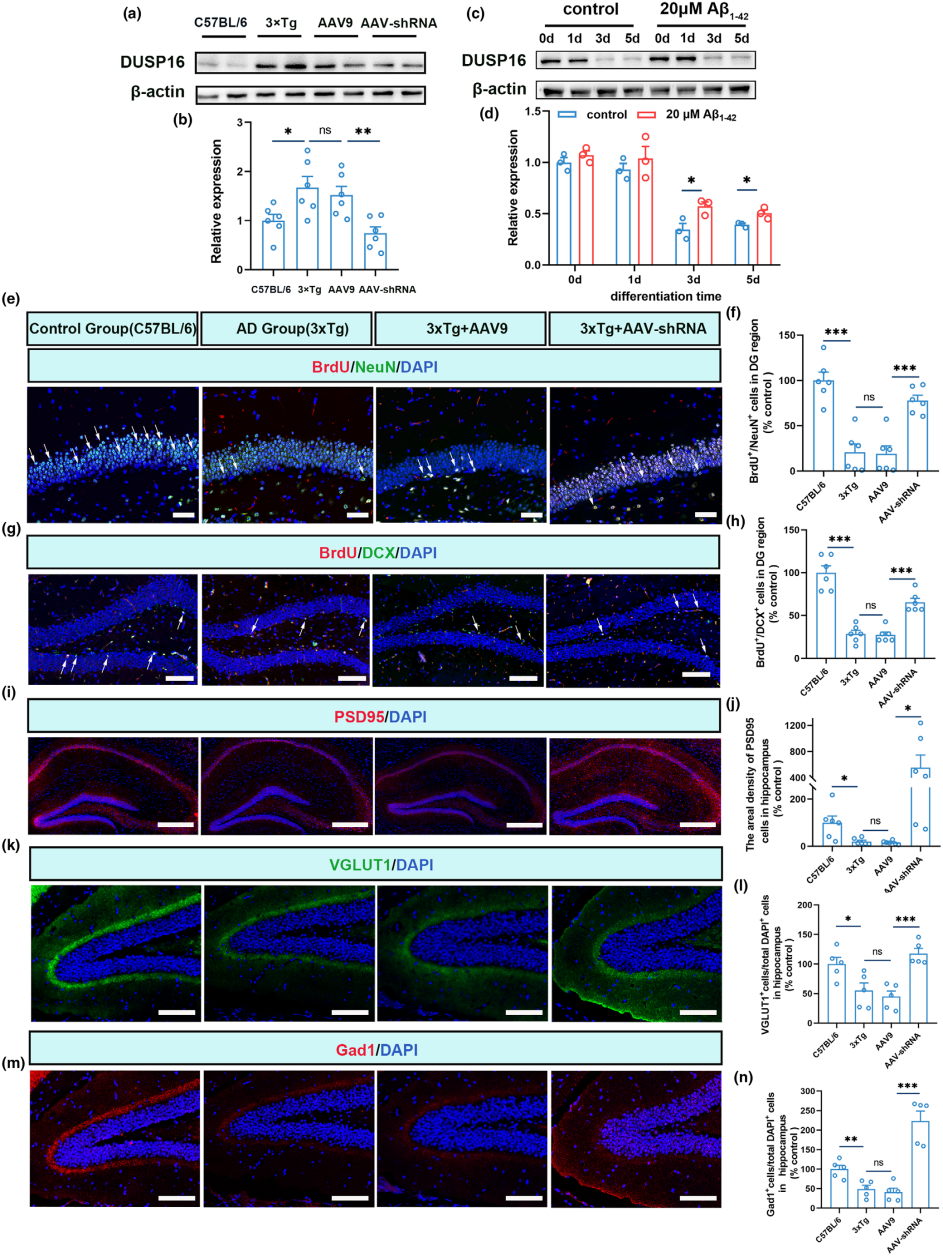
Silencing DUSP16 rescues the impaired neural differentiation of NPCs in AD mice. (a and b) Western blot analyses of DUSP16 in the hippocampal of C57BL/6 and 3xTg mice (*n* = 6). (c and d) Western blot analyses of DUSP16 in Aβ_1‐42_‐treated and blank differentiated C17.2 cells (*n* = 3). (e and f) Sample immunofluorescence images of BrdU^+^/NeuN^+^ cells in the C57BL/6 and 3xTg mice dentate gyrus, and quantitative comparison of BrdU^+^/NeuN^+^ cells in the DG region of C57BL/6 and 3xTg mice (*n* = 6 per group). The 3xTg mice were treated with AAV9 and AAV‐DUSP16, or without treatment. Blue, DAPI; green, NeuN; red, BrdU. Scale bars, 50 μm. (g and h) Sample immunofluorescence images of BrdU^+^/DCX^+^ cells in the dentate gyrus of C57BL/6 and 3xTg mice, and quantitative comparison of BrdU^+^/DCX^+^ cells in the DG region of C57BL/6 and 3xTg mice (*n* = 6 per group). The 3xTg mice were treated with AAV9 and AAV‐DUSP16, or without treatment. Blue, DAPI; green, DCX; red, BrdU. Scale bars, 100 μm. (i and j) Sample immunofluorescence images of PSD95^+^ cells in the hippocampus of C57BL/6 and 3xTg mice (*n* = 6 per group), followed by quantitative analysis of PSD95^+^ cells (j). The 3xTg mice were treated with AAV9 and AAV‐DUSP16, or without treatment. Blue, DAPI; red, PSD95. Scale bars, 200 μm. (k and l) Sample immunofluorescence images of VGLUT1^+^ cells in the hippocampus of C57BL/6 and 3xTg mice (*n* = 5 per group), followed by quantitative analysis of VGLUT1^+^ cells (L). The 3xTg mice were treated with AAV9 and AAV‐DUSP16, or without treatment. Blue, DAPI; green, VGLUT1. Scale bars, 50 μm. (m and n) Sample immunofluorescence images of Gad1^+^ cells in the hippocampus of C57BL/6 and 3xTg mice (*n* = 5 per group), followed by quantitative analysis of Gad1^+^ cells (n). The 3xTg mice were treated with AAV9 and AAV‐DUSP16, or without treatment. Blue, DAPI; red, Gad1. Scale bars, 50 μm. *p < 0.05; **p < 0.01; ***p < 0.001; Student's *t*‐test was used for all data analyses. Data are presented as mean ± SEM.

Initially, the DUSP16 protein expression was assessed by western blot in two AD model mice. Compared to wild‐type mice, elevated DUSP16 expression appeared in both 3xTg and SAMP8 mice (Figure 2a,b; *p* < 0.05, and Figure S1a,b,d–f,h; *p* < 0.05, *t*‐test; *n* = 6). Besides, we assessed DUSP16 protein expression in differentiated C17.2 murine NPCs. After 5 days of neural differentiation, the protein level of DUSP16 was higher in the 20 μM Aβ_1‐42_ treated group compared to the control group (Figure 2c,d; *p* < 0.05), further suggesting a relationship between DUSP16 and NPC neural differentiation.

To investigate whether NPC neural differentiation is affected in AD, we performed immunofluorescence to compare the number of BrdU^+^/DCX^+^(a marker for new immature neurons), BrdU^+^/NeuN^+^(a marker for new mature neurons), and BrdU^+^/GFAP^+^ (a marker for new astrocytes) cells in 3xTg and SAMP8 mice. Both 3xTg and SAMP8 mice had a lower amount of new immature and mature neurons (Figure 2e–h; *p* < 0.001, Figure S2a–d, and Figure S3; *p* < 0.05 for b and *p* < 0.01 for d, *t*‐test; *n* = 6) in the dentate gyrus compared to their control group (C57 and SAMR1 mice). Additionally, there was no significant difference in new astrocytes between 3xTg and C57 mice (Figure S2e,f; *p* > 0.05, *t*‐test; *n* = 6), while SAMR1 mice had a greater number of new astrocytes than SAMP8 mice (Figure S2g,h; *p* < 0.001, *t*‐test; *n* = 6). Moreover, the number of PSD95^+^ cells decreased significantly in AD mice (Figure 2i,j; *p* < 0.05, and Figure S4a,b; *p* < 0.05, *t*‐test; *n* = 6) compared with the control groups. Besides, Pearson's correlation analysis was performed between elevated DUSP16 expression and the neural differentiation of NPCs in two AD mouse models. BrdU^+^/NeuN^+^ positive cells in the hippocampus of two AD mouse models and their controls were selected as markers for NPC neural differentiation. Significant inverse correlations were detected (Figure S4c,d): 3xTg mice (Pearson's correlation = 0.662, *p* = 0.00043), SAMP8 mice (Pearson's correlation = 0.513, *p* = 0.0103).

To explore the role of DUSP16 in regulating neural differentiation of NPCs, the neural differentiation of C17.2 murine NPCs was investigated using immunofluorescence. The number of β3‐tubulin‐positive cells and the synaptic length were measured. After 5 days of differentiation, the percentage of β3‐Tubulin positive cells among total cells and the synaptic length in differentiated C17.2 cells significantly declined in the 20 μM Aβ_1‐42_ treated group (Figure S4e–g; *p* < 0.001 for f and *p* < 0.01 for g, *t*‐test; *n* = 4), which suggests that Aβ_1‐42_ treatment diminishes differentiation efficiency of C17.2 murine NPCs. Taken together, impaired neural differentiation of NPCs correlated with increased DUSP16 in AD mice and C17.2 cells.

### 2.3 Silencing DUSP16 rescues the neural differentiation of NPCs in AD mice

Impaired neural differentiation of NPCs in AD models coincided with elevated levels of DUSP16; whether DUSP16 knockdown could rescue the neural differentiation deficits was immediately investigated in 3xTg and SAMP8 mice. Tail intravenous injection of AAV‐DUSP16 (AAV‐shRNA, 25 μL, PHP.EB) was administered to suppress DUSP16 expression in 4 or 5‐month 3xTg and SAMP8 mice. Meanwhile, control AAV (AAV9, 25 μL, PHP.EB) was injected to the same age 3xTg and SAMP8 mice. The silencing efficiency was respectively 49% and 56% that were evaluated through western blot (Figure 2b and Figure S1I). In the DG region of AAV‐shRNA‐treated 3xTg mice, the quantity of BrdU^+^/DCX^+^, BrdU^+^/NeuN^+^, and PSD95^+^ cells was significantly higher than that in the AAV‐9‐treated mice (Figure 2e–j; *p* < 0.001 for f and h, *p* < 0.05 for j, Figures S2a–d and S4a,b; *p* < 0.05, *t*‐test; *n* = 6). Nevertheless, there was no difference in the amount of BrdU^+^/GFAP^+^ cells between the AAV9 group and the AAV‐shRNA group (Figure S2e–h; *p* > 0.05, *t*‐test; *n* = 6). Moreover, DUSP16 knockdown mice had a higher number of VGLUT1^+^ (Figure 2k,l; *p* < 0.001, and Figure S5a,b; *p* < 0.001, *t*‐test; *n* = 5) and Gad1^+^ cells (Figure 2m,n; *p* < 0.001, and Figure S5c,d; *p* < 0.001, *t*‐test; *n* = 5). Furthermore, we assessed the alternation of NPCs pool by counting Nestin^+^ cells, and there were no significant changes of Nestin^+^ cells in the hippocampus of 3xTg and SAMP8 mice (Figure S5e–h; *p* > 0.05, *t‐*test; *n* = 5). Our data suggest that silencing DUSP16 ameliorates neural differentiation impairments without affecting NPCs pool.

### 2.4 Inhibition of DUSP16 expression alleviates deficient basal transmission and LTP in hippocampal synapses

**FIGURE 3:**
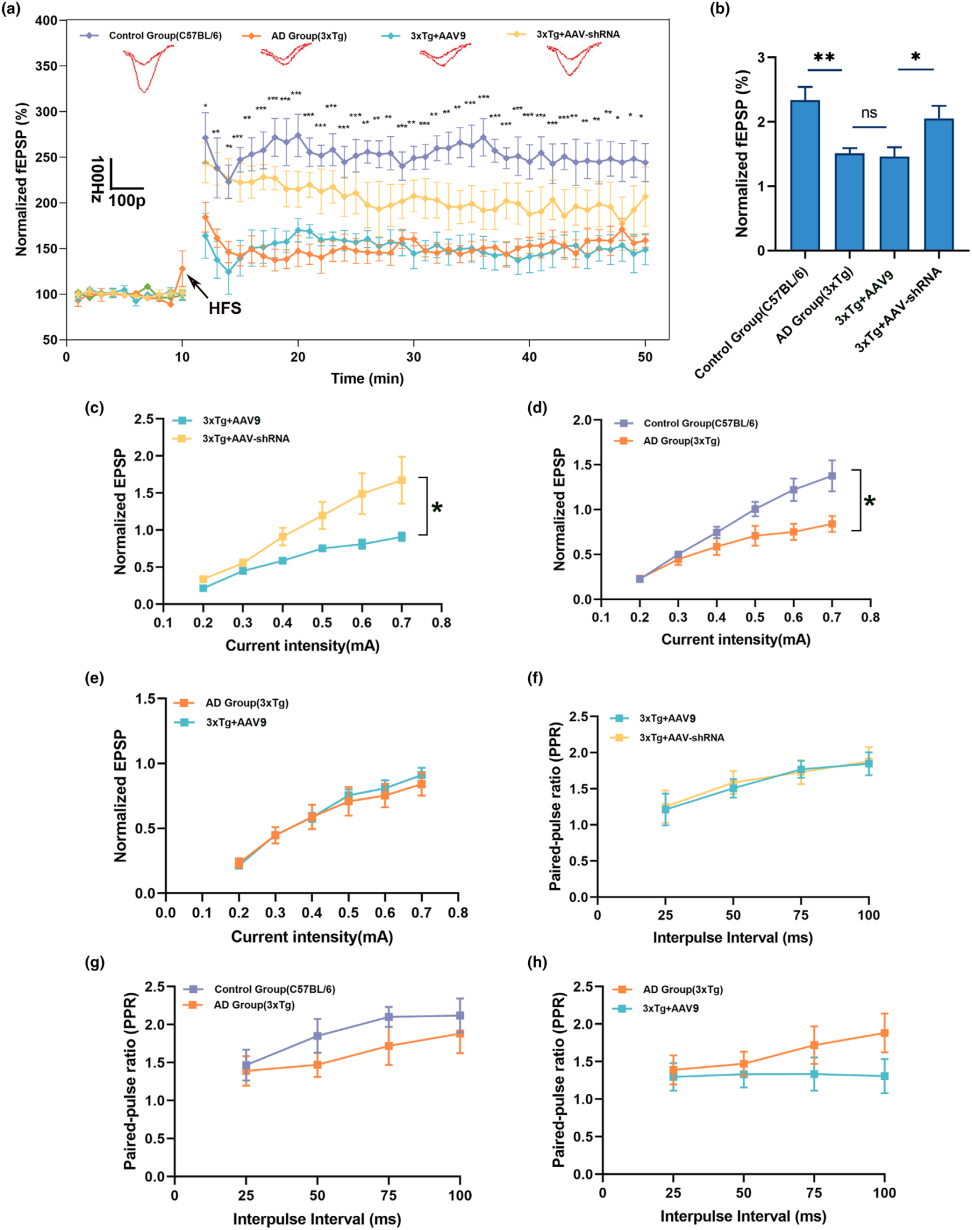
Inhibition of DUSP16 expression alleviates deficient basal transmission and LTP in hippocampal synapses. (a) Normalized fEPSP amplitude to field stimulation before and after HFS in C57 and 3xTg mice treated with AAV‐DUSP16 or AAV9, or without treatment. The slope of the initial component of the fEPSP was normalized to baseline, and a baseline for 10 mins was obtained before HFS. (b) Mean value of potentiation between 12 and 50 min in four mice groups after HFS. (c–e) EPSPs at various intensities were observed in the I/O curve in C57 and 3xTg mice treated with AAV‐DUSP16 or AAV9, or without treatment. (f–h) PPR with different inter‐stimulus intervals in C57 and 3xTg mice treated with AAV‐DUSP16 or AAV9, or without treatment. *n* = 5 mice per group, *p < 0.05; **p < 0.01; ***p < 0.001. One‐way ANOVA was used in (a); Student's *t*‐test was used in (b); two‐way ANOVA was used in (c–h). Data are presented as mean ± SEM.

To investigate whether DUSP16 knockdown improves synaptic function, we then examined alterations in Long‐term potentiation (LTP) and synaptic transmission in AAV‐DUSP16‐treated mice. In 6‐month 3xTg mice, we first assessed hippocampal LTP in the CA3‐CA1 circuit and observed that AAV‐shRNA‐treated mice showed meliorative impairment of LTP, as evidenced by a marked increase in the normalized fEPSP slope compared to AAV‐9‐treated mice (Figure 3a,b; *p* < 0.05, one‐way ANOVA; *n* = 5). In addition, the input and output relationship (I/O) curve was shifted upward in AAV‐shRNA‐treated 3xTg mice (Figure 3c–e; *p* < 0.05 for c and d, two‐way ANOVA; *n* = 5), indicating a strengthening of synaptic transmission. To determine whether this enhancement occurs at pre‐ or postsynaptic sites, we evaluated the paired‐pulse ratio (PPR). No significant changes were detected in PPR between AAV‐shRNA‐treated and AAV‐9‐treated 3xTg mice (Figure 3f; *p* > 0.05, two‐way ANOVA; *n* = 5). Similar results were observed in SAMP8 mice (Figure S6). Taken together, these results imply that the downregulated DUSP16 expression rescues synaptic transmission deficits in both 3xTg and SAMP8 mice.

### 2.5 Inhibition of DUSP16 expression improves memory impairments in AD mice

**FIGURE 4:**
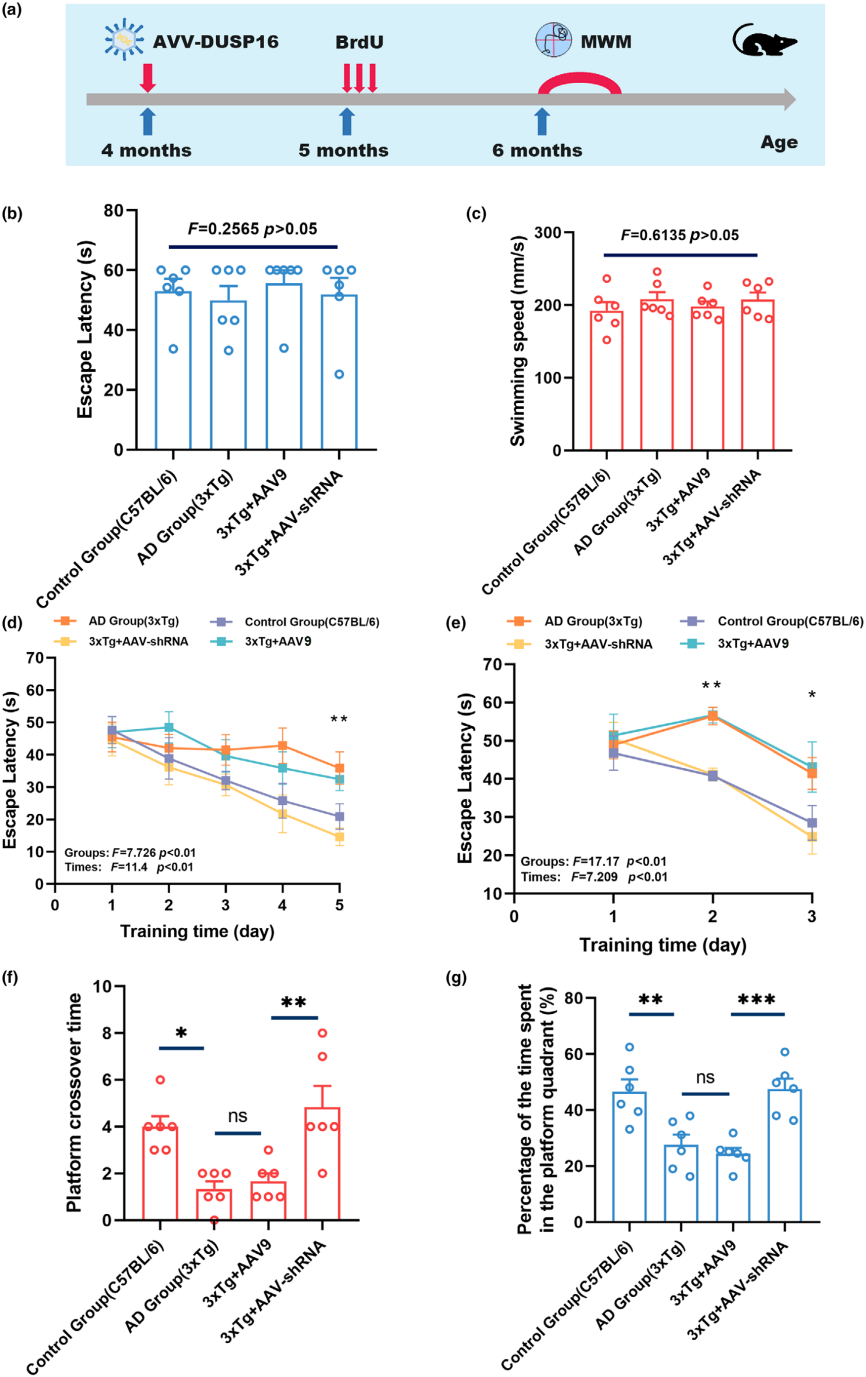
Inhibition of DUSP16 expression improves memory impairments in AD mice. (a) Experimental scheme for assessing cognitive function in C57BL/6 and 3xTg mice treated with AAV‐DUSP16. (b and c) There were no difference among the four mice groups (3xTg) in the escape latency and swimming speed in the visible platform trail. (d) Silencing DUSP16 resulted in the decreased escape latency in 3xTg mice in the hidden platform trail. (e) Silencing DUSP16 resulted in the decreased escape latency in 3xTg mice in the reference trail. (f and g) Silencing DUSP16 resulted in the reduced platform crossover times (f) and percentage of time spent in the platform quadrant (g) in 3xTg mice in the probe trail. *n* = 6 mice per group, *p < 0.05; **p < 0.01; ***p < 0.001. Student's *t*‐test was used in (f and g); one‐way ANOVA was used in (b); Two‐way ANOVA was used in (d and e). Data are presented as mean ± SEM.

Spatial memory of 6‐month mice was evaluated by conducting the Morris water maze (MWM) (Figure 4a). Initially, we confirmed that all mice exhibited no difference in the swimming ability (Figure 4b,c; *p* > 0.05, and Figure S7a,b; *p* > 0.05, *t*‐test; *n* = 6). The hidden platform trail indicated that both different groups and training days had a significant influence on the latency (Figure 4d and Figure S7c,d, two‐way ANOVA; *n* = 6). The AAV‐shRNA‐treated mice displayed shorter latency at the fifth day compared to the AAV9 group (Figure 4d; *p* < 0.01 and Figure S7c; *p* < 0.001, one‐way ANOVA; *n* = 6). During the probe trial, compared with the AAV9‐treated mice, AAV‐shRNA‐treated mice showed significantly increased crossover times (Figure 4f; *p* < 0.001 and Figure S7e; *p* < 0.01, *t*‐test; *n* = 6) and longer latency spent in the platform quadrant (Figure 4g; *p* < 0.01 and Figure S7f; *p* < 0.05, *t*‐test; *n* = 6). The latency was also affected by the two factors (groups and training days) in the last 3 days of acquisition training (Figure 4e and Figure S7h, two‐way ANOVA; *n* = 6). Furthermore, AAV‐shRNA‐treated mice displayed shorten latency on the second and third days than AAV9‐infected mice (Figure 4e; *p* < 0.05 and Figure S7h; *p* < 0.01, one‐way ANOVA; *n* = 6). Similar results were observed in the comparison between AAV‐shRNA‐treated and AAV9‐treated SAMP8 mice (Figure S7). Collectively, these results indicated that decreased DUSP16 expression ameliorated spatial memory deficits in 3xTg and SAMP8 mice.

### 2.6 DUSP16 regulates neural differentiation through the JNK‐SOX2 pathway

**FIGURE 5:**
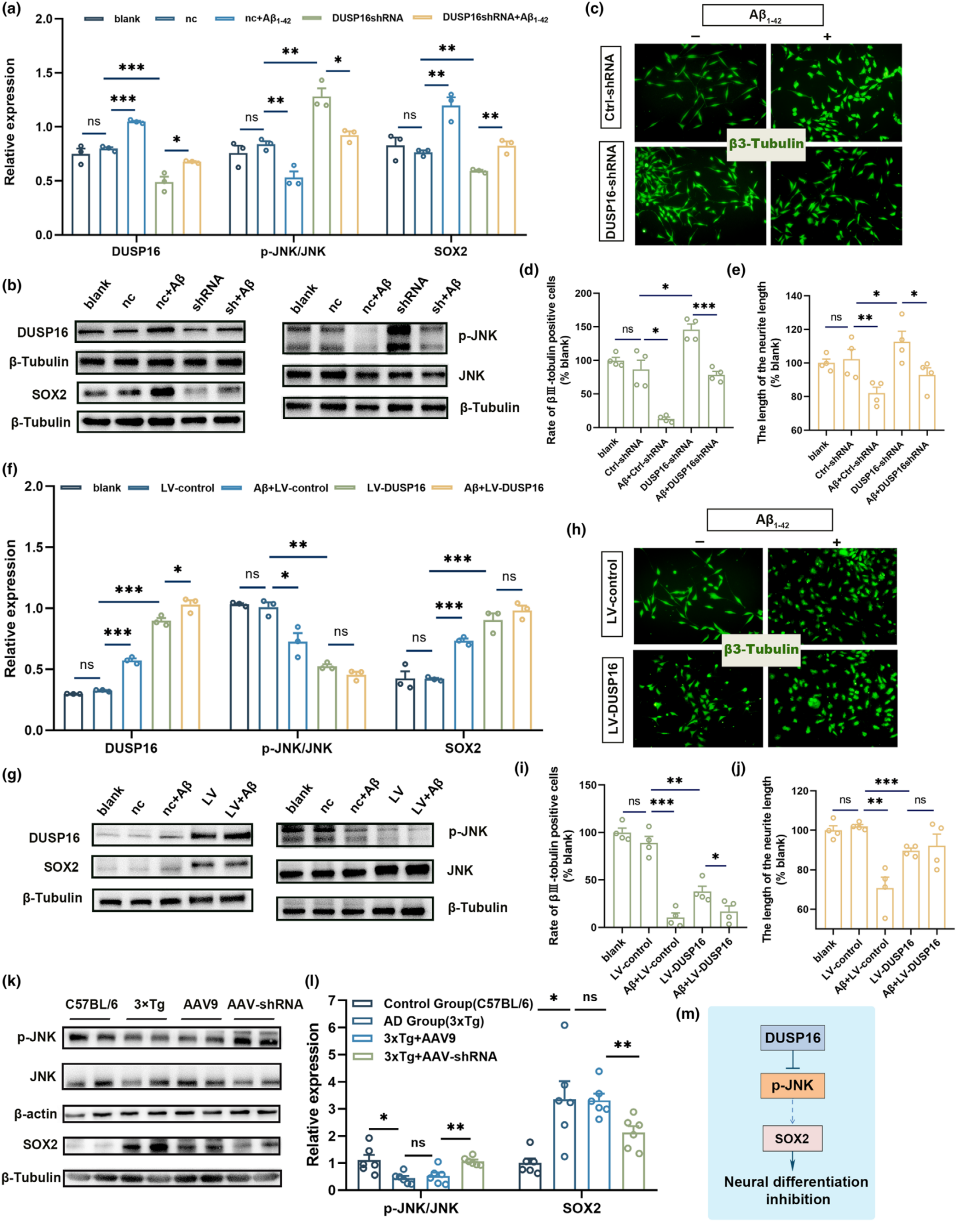
DUSP16 regulates neural differentiation through the JNK‐SOX2 pathway. (a and b) Western blot analyses of DUSP16, SOX2, and p‐JNK/JNK ratio in C17.2 cells with silencing DUSP16 (*n* = 3, normalized to β‐tubulin). (c) Representative images showing that DUSP16‐ShRNA‐treated or Ctrl‐ShRNA‐treated C17.2 cells incorporated β3‐tubulin (green) under differentiating conditions (5d), with or without Aβ_1‐42_ treatment. Scale bar, 100 mm. (d and e) Quantitative analysis showing that DUSP16‐knockdown increased the neural differentiation rate and neurite length of both Aβ_1‐42_‐treated and blank C17.2 cells (*n* = 4). (f and g) Western blot analyses of DUSP16, SOX2, and p‐JNK/JNK ratio in DUSP16 overexpressed C17.2 cells (*n* = 3, normalized to β‐Tubulin). (h) Representative images showing that LV‐DUSP16‐treated or LV‐control‐treated C17.2 cells incorporated β3‐Tubulin (green) under differentiating conditions (5d), with or without Aβ_1‐42_ treatment. Scale bar, 100 mm. (i and j) Quantitative analysis showing that DUSP16 overexpression reduced the neural differentiation rate and neurite length of both Aβ_1‐42_‐treated and blank C17.2 cells (*n* = 4). (k and l) Western blot analyses of p‐JNK/JNK ratio and SOX2 in C57BL/6 and 3xTg mice. The 3xTg mice were treated with AAV9 and AAV‐DUSP16, or without treatment (*n* = 6). (m) Schematic drawing showing that DUSP16 could inhibit neural differentiation of NPCs through JNK signaling. *p < 0.05; **p < 0.01; ***p < 0.001; Student's *t*‐test was used for all data analyses. Data are presented as mean ± SEM.

We analyzed the impacts of DUSP16 knockdown and overexpression on NPC neural differentiation by transferring lentivirus vectors into the cells. The silencing and overexpression efficiencies were 38.7% and 178%, respectively, as determined by western blot analysis (Figure 5a,b,f,g). After 5 days of differentiation, knockdown of DUSP16 alleviated neural differentiation deficits caused by Aβ_1‐42_ treatment (Figure 5c–e; *p* < 0.001 for d, *p* < 0.05 for e, *t*‐test; *n* = 4). Conversely, the decreased neural differentiation was aggravated by DUSP16 overexpression (Figure 5h–j; *p* < 0.05 for i, *t*‐test; *n* = 4). These data suggested a regulatory role for DUSP16 in neural differentiation within C17.2 cells.

The effect of DUSP16 on neural differentiation regulation was further investigated. DUSP16 is implicated in the MAPK pathway through dephosphorylating JNK and P38 (Tanoue et al., 2001). Therefore, the phosphorylation level of JNK and P38 was measured in the DUSP16‐silencing NPCs, and the data showed that there was no change in the expression level of P38 phosphorylation or total P38 (Figure S8a,b; *p* > 0.05, *t*‐test; *n* = 3). To further validate the binding relationship of DUSP16 and JNK, the immunofluorescence colocalization experiments were conducted in hippocampus tissue of SAMP8 mice, confirming that DUSP16 directly binds to JNK (Figure S8c). SOX2 has been identified as a determining factor of neural differentiation (Cui et al., 2018). Thus, we investigated whether JNK may influence neural differentiation through SOX2. The exposure of Aβ_1‐42_ led to decrease the ratio of p‐JNK/JNK and subsequently induced SOX2 in NPCs (Figure S8d–h; *p* < 0.05 for e and f, *t*‐test; *n* = 3). In DUSP16‐silencing differentiated cells, p‐JNK/JNK ratio was upregulated while SOX2 expression was reduced (Figure 5a,b; *t*‐test; *n* = 3); In DUSP16‐overexpressed C17.2 cells, the reduction in p‐JNK/JNK ratio was observed, while the level of SOX2 was restored (Figure 5f,g; *t*‐test; *n* = 3). In addition, the in vitro data were further verified by animal experiments. In the AD group (3xTg), the ratio of p‐JNK/JNK was significantly decreased (Figure 5k,l; *p* < 0.01, *t*‐test; *n* = 6) while the level of SOX2 was increased compared to the control group (C57BL/6 and SAMR1) (Figure 5k,l, and Figure S1a–c; *p* < 0.05, *t*‐test; *n* = 6). Meanwhile, the AAV‐shRNA group had a higher p‐JNK/JNK ratio and a lower SOX2 expression than the AAV9 group (Figure 5k,l, and Figure S1a–c; *p* < 0.001, *t*‐test; *n* = 6), suggesting that silencing DUSP16 in 3xTg mice may result in upregulated p‐JNK and reduced expression of SOX2. The similar data were observed in SAMP8 mice (Figure S1d–i). Besides, the role of DUSP16 in Aβ deposition was investigated. In 6‐month‐old 3xTg mice, a greater number of Aβ plaques appeared in the hippocampus compared to C57 mice (Figure S9c,d; *p* < 0.05, *t*‐test; *n* = 3), while no significant change of Aβ deposition was found in SAMP8 and SAMR1 mice (Figure S9a,b; *p* > 0.05, *t*‐test; *n* = 3). After DUSP16 knockdown, there was no obvious difference in Aβ deposition between the AAV‐shRNA group and the AAV9 group (Figure S9a–d), which suggested that DUSP16 inhibition may not influence Aβ deposition in 3xTg and SAMP8 mice. Consequently, DUSP16 participated in the neural differentiation of NPCs through the JNK‐SOX2 pathway.

### 2.7 ELK1 involves in DUSP16 transcriptional activation

**FIGURE 6:**
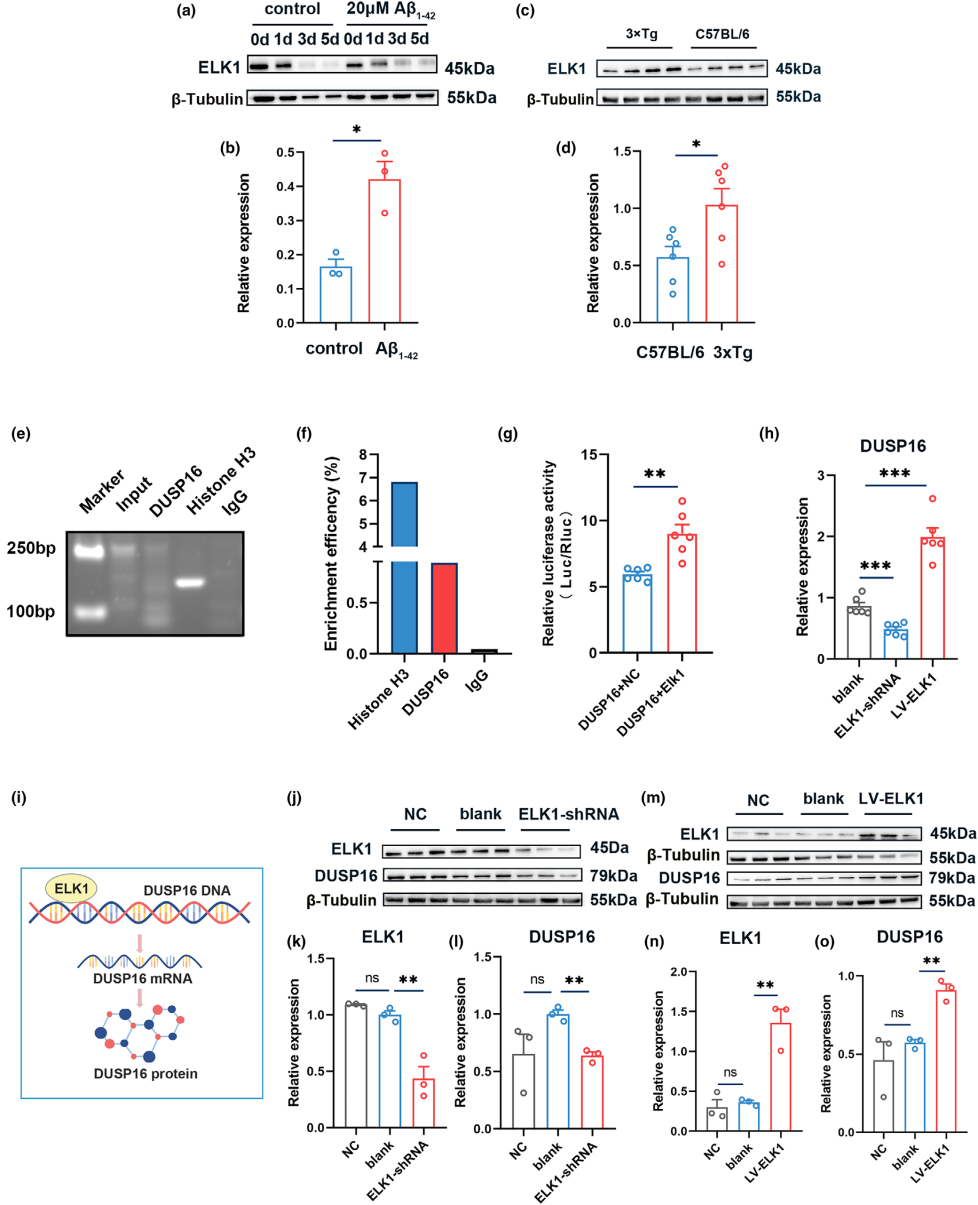
ELK1 involves in DUSP16 transcriptional activation. (a and b) Western blot analyses of ELK1 in Aβ_1‐42_‐treated and blank differentiated C17.2 cells (*n* = 3). (c and d) Western blot analyses of ELK1 in the hippocampus of 3xTg and C57BL/6 mice (*n* = 3). (e) DNA gel electrophoresis diagram of ChIP‐qPCR products. (f) ELK1 ChIP‐qPCR analyses in the positive control group (Histone H3), the target gene group (DUSP16) and the negative control group (IgG). (g) Relative luciferase activity of NC and ELK1 vectors in the 293 T cells transfected with pGL3‐Basic‐Dusp16_Pro reporter vectors. (h) Quantification analyses of DUSP16 mRNA in ELK1‐shRNA and LV‐ELK1 C17.2 cells (*n* = 3). (i) Schematic drawing showing that ELK1 binds to the DUSP16 promoter so that can regulate the expression of DUSP16 mRNA and protein. (j–l) Western blot analyses of ELK1 and DUSP16 in NC, blank and ELK1‐downregulated C17.2 cells (*n* = 3). (m–o) Western blot analyses of ELK1 and DUSP16 in NC, blank and ELK1‐overexpressed C17.2 cells (*n* = 3). Gapdh was used as the internal control for quantitative PCR analysis. *p < 0.05; **p < 0.01; ***p < 0.001; Student's *t*‐test was used for all data analyses. Data are presented as mean ± SEM.

The investigation into the mechanism underlying the elevation of DUSP16 in AD further explored the relationship between increased DUSP16 expression and its upstream transcription factors. The promoter regions of DUSP16 were obtained from the NCBI database (http://www.ncbi.nlm.nih.gov), and the binding transcription factors of DUSP16 promoter regions were predicted using the PROMO database (https://alggen.lsi.upc.es/cgi‐bin/promo_v3/promo/promoinit.cgi?dirDB=TF_8.3). Hence, ELK1 was predicted to be the crucial mediator for the DUSP16 gene. Furthermore, the level of ELK1 protein was performed by western blot in the differentiated C17.2 murine NPCs and AD model mice. After 3 and 5 days of Aβ_1‐42_ treatment, the ELK1 level was higher than the control group (Figures 6a,b and S10a,b; *p* < 0.05, *t*‐test; *n* = 3). In both 3xTg and SAMP8 mouse models, ELK1 protein expressions increased compared to their respective control groups (Figure 6c,d and Figure S10c,d; *p* < 0.05, *t*‐test; *n* = 6). The ELK1 expression level decreased gradually with prolonged differentiation time, paralleling the changes in DUSP16 expression. Therefore, we assumed that the increased expression of the transcription factor ELK1 might involve in the upregulation of DUSP16 under AD conditions.

To validate the above hypothesis, the ChIP‐qPCR analysis was conducted to investigate whether ELK1 engaged in DUSP16 transcription. In line with the prediction of the PROMO database, one putative ELK1 binding site were chosen in the promoter sequence of DUSP16, and synthetic primers were designed for ChIP‐qPCR analysis (Table S3). The ChIP‐qPCR results confirmed that ELK1 binds to the DUSP16 promoter region. The enrichment efficiency of the DUSP16 promoter was 0.89% (the quality control requirement is 0.1% ~ 1%) (Figure 6e,f), which was qualitatively confirmed by follow‐up PCR assay.

Subsequently, to further validate whether ELK1 can transactivate its target gene DUSP16, dual‐luciferase reporter (DLR) gene assays were carried out by constructing overexpress‐ELK1 vector and DUP16 promoter‐luciferase reporter plasmids. Compared to the negative control vector, ELK1 overexpression significantly elevated relative luciferase activity after the co‐transfection of DUSP16 promoter plasmids (Figure 6g; *p* < 0.01, *t*‐test; *n* = 6), which indicated that ELK1 might transactivate DUSP16 promoter.

Next, to detect the alterations of DUSP16 mRNA and protein, stable C17.2 cell lines of silencing and overexpressing ELK1 were established. The efficiency of silence and overexpression were 47.5% and 162% (Figure S10e), respectively, as verified by RT‐qPCR and western blot. Following the decreased expression of ELK1, DUSP16 mRNA and protein levels were significantly reduced in the proliferative C17.2 cells (Figure 6h–l; *p* < 0.001 for h, *p* < 0.01 for l, *t*‐test; *n* = 6 for h, *n* = 3 for l). Conversely, the enhanced expression of DUSP16 mRNA and protein were observed in the overexpressed ELK1 cells (Figure 6h,m–o; *p* < 0.001 for h, *p* < 0.01 for o, *t*‐test; *n* = 6 for h, *n* = 3 for o). Overall, these findings above implied that ELK1 was involved in DUSP16 transcriptional regulation, which subsequently lead to the increased DUSP16 expression observed in AD.

## 3 DISCUSSION

Here, the analysis of clinical SNPs data from ADNI revealed a potential association between DUSP16 and the risk of transitioning from MCI to AD dementia. The alternations of neurogenesis are observed in various stages of AD disease progression, while it has been implied that DUSP16 was involved in neurogenesis (Moreno‐Jiménez et al., 2019; Zega et al., 2017). Elevated DUSP16 was observed in both 3xTg and SAMP8 mice, accompanied by impairments in hippocampal neurogenesis. Silencing DUSP16 promoted neural differentiation of NPCs, increased the number of newly born neurons, and ameliorated synaptic transmission and cognitive function. Furthermore, the upregulated DUSP16 may be relevant to the increased expression of ELK1 in AD. Our findings suggested that the stimulation of neural differentiation by DUSP16 knockdown in NPCs could improve cognitive function through supplying the new‐born neurons and enhancing synaptic transmission in AD mice.

DUSP16 has recently gained attention for its role in neural differentiation and neurite outgrowth (Maor‐Nof et al., 2016; Zega et al., 2017). Building upon these findings, we hypothesized that DUSP16 might be involved in cognitive function by regulating neural differentiation of NPCs. DUSPs selectively dephosphorylate threonine and tyrosine residues on MAPKs, resulting in their deactivation (Jeffrey et al., 2007). Generally identified as a negative regulator of P38 and JNK, DUSP16 exhibits different inactivation selectivity depending on the tissue/organ and cell type (Lee et al., 2010; Low et al., 2021; Tanoue et al., 2001). Our current study demonstrated that DUSP16 triggers JNK inactivation through dephosphorylation, rather than affecting P38 in NPCs. JNK signaling plays a pivotal role in regulating neural differentiation through its interactions with diverse transcription factors (Zeke et al., 2016). Notably, JNK may be involved in the neural differentiation process of NPCs by negatively regulating FOXO1 (Deng et al., 2011; Xu et al., 2011), a crucial regulator of neural stem cell differentiation (Kim et al., 2015). FOXO1 could directly bind to SOX2 regulatory region and induce upregulation of the NPC‐specific biomarkers (Zhang et al., 2011), which inhibited neuronal differentiation. In summary, our observations indicate that increased expression of DUSP16 in AD mice leads to JNK inactivation and subsequent induction of SOX2 expression, ultimately impairs neural differentiation of NPCs (Figure S11). Previous transcriptomic investigations alongside our own results confirm that increased DUSP16 expression in AD mice. A critical question has emerged as to why DUSP16 expression was elevated in AD mice. Our finding demonstrated that ELK1 involved in DUSP16 transcriptional activation. Previous findings have reported that no obvious alteration of total ELK1 expression was occurred in vitro following Aβ_1‐42_ treatment, alongside nuclear Elk‐1 phosphorylation level remained unchanged in hippocampal slices of AD mice (Liu et al., 2019; Szatmari et al., 2013). Nevertheless, our study identified changes in total ELK1 expression in C17.2 cells and AD model mice. It has been indicated that phosphorylation might not be essential for ELK1. ELK1 can bind directly and independently of phosphorylation to CREB‐binding protein (CBP). Subsequently, the C‐terminal transactivation domains interact with the N‐terminus of CBP (Buchwalter et al., 2004). Furthermore, our luciferase receptor assay results suggest that ELK1 can transcriptionally activate the DUSP16 promoter without the need for phosphorylation. It has been indicated that phosphorylation might not be essential for ELK1. To sum up, the transcriptional activation of ELK1 for DUSP16 may not rely on its phosphorylation under AD conditions, but rather on enhancing its own expression, warranting further investigation into the reasons behind this divergence. Maintaining a lifelong pool of NPCs is crucial for AHN (Gillotin et al., 2021). Given that silencing DUSP16 enhances NPC differentiation, it remains critical to determine whether this leads to the depletion of the NPCs pool, impacting the translational potential of this intervention approach. Our findings indicated that there were no noticeable alterations in NPCs of AD mice. This aligns with the observation that deficiency in DUSP16 can actually expand the neural progenitor pool (Zega et al., 2017). Consequently, our identification of boosted NPC differentiation through silencing DUSP16 in the brains of AD mice without obvious NPCs pool depletion implies the potential involvement of DUSP16 in governing NPC proliferation and differentiation.

Our findings demonstrate that the enhanced neural differentiation of NPCs correlates with DUSP16 inhibition, whereas the characteristics of newborn neurons remain largely unexplored. The glutamatergic and GABAergic systems serve as the most important parts of hippocampal neural network (Cheng et al., 2021). Glutamatergic neurons, particularly those involving N‐methyl D‐aspartate receptor (NMDAR) activation, play a crucial role in long‐term synaptic plasticity and cognitive function (Pinky et al., 2023). Whereas GABAergic neurons are essential for facilitating an accurate encoding of information in the brain, mainly by restricting the temporal window for excitatory synaptic inputs integration and leading to spike generation, which are implicated in complex cognitive functions (Pouille & Scanziani, 2001). Therefore, changes in the quantity of glutamatergic and GABAergic neurons were detected in the present study. A loss of both neuron types was observed in ourAD mouse models, consistent with findings of GABAergic and glutamatergic neuron deficits in the brains of AD patients (Melgosa‐Ecenarro et al., 2023; Rodriguez‐Perdigon et al., 2016). After DUSP16‐knockdown, the increased number of glutamatergic and GABAergic neurons were found in AD model mice; the increased neurons probably originate from NPCs located in the hippocampus. Notably, the increase in GABAergic neurons surpassed that of glutamatergic neurons. There are several possible reasons for explaining this phenomenon. First, given that growing evidence demonstrated that aberrant glutamatergic system activation and GABAergic dysfunction resulted in neuronal hyperexcitability and degeneration in the AD brain (Bi et al., 2020), the enlarged GABAergic neurons could be a compensatory mechanism against hyperexcitability. Second, GABAergic neurons appear to be relatively spared during the pathogenesis of AD (Govindpani et al., 2017), which may enhance their survival prospects under AD conditions.

Adult neurogenesis occurs primarily in two key neurogenic regions: the subgranular zone (SGZ) of the hippocampal dentate gyrus and the subventricular zone (SVZ) of the lateral ventricles (Salta et al., 2023). Neurogenesis in the SVZ produces olfactory bulb interneurons, which may influence cognitive processes indirectly (Lim & Alvarez‐Buylla, 2016). Additionally, while neuroblasts migrate from the SVZ to the olfactory bulb (OB) via the rostral migratory stream (RMS) in rodent models (Zhao et al., 2008), the existence and configuration of the adult RMS in humans remain highly debated (Arellano & Rakic, 2011; Culig et al., 2022; Sanai et al., 2007). Specifically, Sanai et al. identified that RMS exists in human newborns but is absent by the age of 18 months. Contrarily, Curtis et al. reported detecting an RMS in adult humans (Curtis et al., 2007). In comparison, hippocampal neurogenesis has received more extensive examination, with studies on rodents demonstrating that enhanced hippocampal neurogenesis positively impacts cognition in AD models (Salta et al., 2023; Walgrave et al., 2021). Thus, based on this understanding, we chose to focus on exploring the potential role of DUSP16 in hippocampal neurogenesis.

In our study, in addition to transgenic model 3xTg, non‐transgenic model SAMP8 was also selected as in vivo AD mice model. Current research classified AD as familial AD (fAD) and sporadic AD (sAD) in accordance with different pathologies (Zhang, Chen, et al., 2020). 3xTg mice, commonly found in existing transgenic mouse lines, are frequently employed to characterize fAD (Drummond & Wisniewski, 2017). SAMP8 mice, exhibiting typical AD pathologies associated with aging, serve as a model for studying sAD (Liu et al., 2020). In this study, the SAMP8 mice had a higher level of newly born mature neurons than the 3xTg mice (40.9% vs. 20.8%, compared to the respective control group). This finding might relate to neurogenesis defects present in 3xTg mice from early postnatal stages (Liu et al., 2023). Similarities emerged in the number of newly born immature neurons and synaptic plasticity between both mouse models. Notably, compared to their respective controls, Aβ deposition increased markedly in the 3xTg group, while SAMP8 mice showed minimal changes (Figure S9). Particularly relevant to the present study, Aβ deposition was detected at 6 months (Liu et al., 2020) or 8 months age of SMAP8 mice whereas the appearance of Aβ deposition was observed in 3xTg mice aged 4 months (Pallas et al., 2008). However, in DUSP16 knockdown AD mice, no discernible changes were observed in these outcomes between the two model mice. Clinical data analysis indicated that the existence of the specific DUSP16 SNPs were related to the conversion from MCI to AD dementia. A retrospective cohort study involved MCI patients whose mean time to AD conversion was 1.8 years of the baseline PET scan (Pagani et al., 2017). According to this study, the selected conversion time point was within 18 months. Besides, patients with MCI who had high levels of Aβ deposition were more susceptible to developing AD (Villemagne et al., 2013). Consequently, the influence of DUSP16 SNPs on Aβ deposition was investigated further, revealing no significant effects. Besides, DUSP16 inhibition did not appear to impact Aβ deposition in either the 3xTg or SAMP8 mice, suggesting that the association between DUSP16 SNPs and MCI‐to‐AD conversion risk may be relevant to the impact of DUSP16 on hippocampal neurogenesis rather than its role in amyloid pathology.

Our research primarily focuses on the elevation of DUSP16 in AD and its potential connection to upstream transcription factors, specifically ELK1. However, the verification of ELK1's binding site to the DUSP16 promoter region remains unconfirmed. Analysis utilizing the ADNI database reveals a relationship between DUSP16 SNPs and clinical progression in individuals with MCI. Nonetheless, additional samples from MCI to AD patients will be necessary. Another open aspect is to specific knockout of DUSP16, as generating AAV with an NPCs‐specific promoter through stereotaxic injection to the brain would be more appropriate method. Further, to precisely label the type of newborn neurons, BrdU co‐staining alongside markers for glutamatergic and GABAergic neurons will be applied in future study. Finally, future studies are needed to better understand functional consequences of newborn neurons, such as the impact of silencing DUSP16 on synaptic plasticity.

In conclusion, AD pathology triggers an increase in DUSP16, which ultimately hinders AHN. Our findings endorse two mouse models of AD where the DUSP16 suppression serves as an intervention that contributes to cognitive improvement. Recently, the role of small molecules in stimulating neurogenesis through MAPK pathway activation has emerged as a promising therapeutic application in AD. Delineating the molecular mechanisms underlying the role of DUSP16 in neural differentiation may provide impetus for identifying promising strategies to leverage AHN in AD.

## 4 MATERIALS AND METHODS

### 4.1 Study design

This investigation sought to explore the role of DUSP16 in neural differentiation of adult NPCs and to evaluate whether silencing DUSP16 could enhance NPC neural differentiation, leading to the restoration of hippocampal neurogenesis and cognitive deficits in AD. The study encompassed the analysis of clinical data derived from the ADNI database, alongside in vivo animal experiments and in vitro cell culture experiments. To assess the association between DUSP16 SNPs and the progression from MCI to AD dementia, a subset of participants was selected from the ADNI database, including 281 cognitively normal subjects, 480 individuals with MCI, and 48 AD patients. Animal experiments were conducted to assess the impacts of silencing DUSP16 on hippocampal neurogenesis, synaptic transmission, and cognitive disorders using both transgenic mouse models (3xTg) and non‐transgenic mouse models (SAMP8). The impact of DUSP16 on neural differentiation of C17.2 NPCs was analyzed through immunofluorescence (IF) assays. The signaling pathway involved in neural differentiation was investigated using Western blot, RT‐qPCR, CHIP‐qPCR, and dual luciferase reporter assays. The assessment utilized a combination of behavioral tests, IF staining of brain tissues, and electrophysiology (*n* = 5 to 6 mice per group).

### 4.2 ADNI cohort

The ADNI database study commenced in 2003 under the leadership of Weiner et al. It comprised data derived from a long‐term multicenter clinical trial. The enrolled subjects underwent various diagnostic procedures, such as cognitive function assessments, magnetic resonance imaging (MRI) scans, positron emission tomography (PET) scans, gene sequencing, and biomarker assessments, conducted at multiple time points (Veitch et al., 2019). The cohort consisted of 809 participants with available whole genome sequencing data. Participants were categorized into three groups based on their baseline diagnosis: an AD group (*n* = 48), a mild cognitive impairment (MCI) group (*n* = 281), and a normal control group (*n* = 480). There were no significant differences in the gender ratio (*p* > 0.05, Fisher's test) or age (*p* > 0.05, ANOVA test) across the three groups. Table S1 outlines the relevant demographic data of the participants. The whole genomes of all enrolled subjects were sequenced using Illumina's non‐CLIA laboratory, achieving a rough coverage of 30–40×. The obtained sequencing data was analyzed using GATK software (Broad Institute) to pinpoint SNP sites.

### 4.3 Animals and animal grouping

This study used two AD model animals, SAMP8 and 3xTg mice. Male SAMP8 and SAMR1 mice were obtained from the Beijing HFK Bioscience Corporation (Beijing, China). Male 3xTg and C57B/L6 mice were provided by Youdu Biotechnology (Wuhan, China). The 3xTg and SAMP8 mice were respectively divided into three groups: the AD group, the 3xTg/SAMP8 + AAV9 group, and the 3xTg/SAMP8 + AAV‐shRNA group (*n* = 5 or 6 for each group). The age‐matched SAMR1 and C57B/L6 mice were used as normal controls.

### 4.4 AAV transfection

The adeno‐associated virus (AAV) contained a DUSP16 interfering sequence (CAATCAGAAGGTGGTAGTTTA) with AAV9 (AAV‐ShRNA) or AAV9 alone (AAV9), which were generated by Genomeditech Biotechnology (Shanghai, China). Male 3xTg and SAMP8 mice aged 4 or 5 months were injected with 25 μL of AAV9 (1 × 10^13^ viral genome/mL) containing the DUSP16 interfering sequence or empty AAV9 through the caudal vein.

### 4.5 BrdU administration

Five‐month‐old AD mice were administered 100 mg/kg of BrdU (Sigma‐Aldrich, B5002) via intraperitoneal (i.p.) injection daily for 5 days to label new mature neurons (Li et al., 2016). The brains were harvested 28 days after the final BrdU injection. To characterize new immature cells, 6‐month‐old mice were injected with 100 mg/kg of BrdU four times on the day before the mice were killed; the brain extraction occurred 24 h post the final injection.

### 4.6 Immunofluorescence staining

Mouse brain samples were fixed in 4% PFA buffer. The paraffin‐embedded brain slices measured 4 μm in thickness. The tissue samples were deparaffinized with xylene, followed by ethanol washing. Then the antigen repair step was performed, where the tissue sections were immersed in EDTA antigen repair buffer (pH 8.0). To enhance the availability of epitopes for BrdU, the slides were incubated in HCL solution to denature DNA. To block nonspecific binding, the samples were incubated in TBS solution containing 3% BSA for 30 min prior to overnight incubation with primary antibodies at 4°C. Subsequently, sections were incubated with secondary antibodies at room temperature for 1 h. Finally, the stained samples were visualized using a NIKON ECLIPSE C1 immunofluorescent microscope. The following antibodies were used: rabbit anti‐BrdU (Abcam, 1:50), rabbit anti‐GFAP (Servicebio, 1:50), rabbit anti‐PSD95 (AiFang biological, 1:50), rabbit anti‐NeuN (AiFang biological, 1:500), rabbit anti‐Nestin (Abcam, 1:3000), rabbit anti‐DCX (proteintech, 1:4000), rabbit anti‐VGLUT1 (Abcam, 1:4000), rabbit anti‐Gad1 (AiFang, 1:2500), rabbit anti‐Amyloid‐β (AiFang, 1:30), CY3 Goat Anti‐Rabbit IgG (AiFang biological, 1:150).

For cell immunofluorescence staining, cells were cultured in a 96‐well plate. Then fixed with pre‐cooled methanol at −20°C for 5 min, followed by two PBS washes, then infiltrated with 0.25% Triton X‐100 for 10 min at room temperature. Afterwards, the samples were blocked with an immunostaining sealer (Beyotime) for 30 min. The samples were then incubated with an anti‐β3‐Tubulin antibody (Abcam, 1:50) at 4°C overnight. After that, the samples were washed three times with 0.2% PBST and subsequently incubated at room temperature for 1 h with an Alexa Fluor 488 secondary antibody (1:500, Cell Signaling Technology). Subsequently, cells were counterstained with DAPI (Beyotime) for 10 min to visualize the entire cells. Finally, all images were captured using an inverted fluorescence microscope (Olympus, IX53).

### 4.7 Neuronal differentiation efficiency evaluation of C17.2 cells

C17.2 mouse NPCs were purchased from Bnbio Biotechnology (Beijing, China). C17.2 cells were seeded into a 25 cm^2^ cell culture bottle containing proliferation medium (high glucose DMEM supplemented with 10% FBS, 100 U penicillin/mL, and 100 μg streptomycin/mL) for 24 h. Subsequently, the cells were transferred to a 96‐well plate containing differentiation medium (DMEM supplemented with 2% horse serum) for 1, 3, and 5 days. The neuronal‐specific marker β3‐tubulin was used to identify differentiated neurons, which were calculated in irregularly selected non‐overlapping fields under a fluorescence microscope (Olympus). Cells with a neurite extension that are at least twice the diameter of their cell body were defined as the differentiated cells. The neural differentiation was calculated as the ratio of the amount of the differentiated cells to the total number of cells stained with DAPI.

### 4.8 In vivo electrophysiology recordings

The brain slices were prepared from frozen, oxygenated artificial cerebrospinal fluid (ACSF). 3–6 pieces of 400 μm thick coronal brain slices, containing the classic layer of the hippocampus, were retained. Standard borosilicate glass capillary tubes measuring 10 cm in length, with an outer diameter of 1.5 mm and an inner diameter of 0.86 mm, which were drawn using a P‐97 horizontal drawing instrument and divided into four steps to create glass microelectrodes for recording.

Each glass microelectrode was filled with 2 M NaCl to ensure a water entry impedance at the electrode tip ranging from 4 to 6 MΩ. EPSPs were recorded by stimulating the Schaffer collateral pathway via SEN‐3301 and recording in the dendrites of the CA1 radiatus layer. Each stimulation lasted 15 s, followed by a 0.1 ms interval. The stimulation intensity varied from 0.1 to 1.0 mA, with signal recording conducted using an Axoclamp 2B amplifier. Baseline recordings were performed using a single pulse (4 pulses per minute with a waveform width of 0.1 ms) and continued for 15 min before collecting the relevant data. The I/O relationship curve assessed changes in synaptic transmission. Different EPSP slopes were induced by applying a series of different stimulus intensities ranging from 0.2 to 0.7 mA. The I/O curves were obtained by plotting the stimulus intensity on the abscissa and the EPSP slope at different stimulus intensities on the ordinate. Paired‐pulse facilitation (PPF) refers to the facilitation of two consecutive EPSPs with different intervals of 25–100 ms, while PPR was calculated using the amplitude of the second EPSP divided by the amplitude of the first EPSP. PPR variations were inversely proportional to the presynaptic transmitter release. After high‐frequency stimulation (HFS) at 100 Hz, EPSPs were recorded for 60 min to induce LTP. Successful LTP induction was considered when the percentage (%) of EPSP slope change before and after HFS exceeded 20% at 50–60 min.

### 4.9 Western blot analysis in brain tissues and cells

The mice hippocampus and the 12‐well culture plate cells were homogenized and lysed in RIPA lysis buffer (Beyotime Biotechnology, Shanghai, China) for 5 min. Then, they were ultrasonicated and centrifuged. Proteins obtained from the tissue and cells were quantified using a BCA protein assay kit (YEASEN Biotechnology). Subsequently, the proteins were denatured by heating them at 95°C for 5 min in a loading buffer (YEASEN Biotechnology). Proteins were separated on 10% SDS‐polyacrylamide gel electrophoresis and then transferred to polyvinylidene difluoride membranes (Millipore, Burlington, MA, USA). After blocking with blocking buffer (Beyotime Biotechnology, Shanghai, China) for 3 h, sequentially incubated with primary antibodies overnight at 4°C, including rabbit anti‐β‐actin (1:3000), rabbit anti‐β‐tubulin (1:3000), rabbit anti‐DUSP16 (1:1000), rabbit anti‐p‐JNK (1:1000), rabbit anti‐JNK (1:1000), rabbit anti‐p‐p38 (1:1000), rabbit anti‐p38 (1:1000), rabbit anti‐SOX2 (1:1000) from Cell Signaling Technology (CST) as well as rabbit anti‐ELK1 (1:1000) from Abcam. Following washing with TBST, the membrane was incubated at room temperature for 1 h with a secondary antibody, goat anti‐rabbit IgG conjugated with horseradish peroxidase (1:3000; YEASEN Biotechnology). The membrane was detected using ECL western blotting detection reagents (Tannon Science & Technology, Shanghai, China). The ImageJ software was used for protein quantification.

### 4.10 Morris water maze

Morris water maze performance was assessed in a circular water bath maintained at a temperature ranging from 18°C to 22°C, aiming to evaluate the spatial memory of mice. Edible melanin or antholeucin was added to the water to accurately determine the mouse location. The tracks of the mice in the water were recorded using a behavioral instrument.

On the first day, the mice underwent the visible platform trial to assess their ability to locate a 1 cm platform above the water surface within 60 s, ensuring that all mice had an equal swimming ability. From the second to the sixth day, mice participated in the spatial memory trial. Each mouse received two training sessions daily, focusing on locating a platform concealed 1 cm underwater within 60 s. Upon discovering the platform, or when the time elapsed, each mouse remained on the platform for 10 s to reinforce spatial memory. On the sixth day, a probe trial was conducted. Two indicators needed to be recorded: the percentage of time spent in the target quadrant and the mice crossed the platform region times. During the final 3 days of training, mice practiced finding a platform positioned in a different quadrant each day, starting from the opposite quadrant. Each mouse was tested twice a day, and the latency was analyzed.

### 4.11 Lentiviral transfection

C17.2 cells were infected with lentivirus carrying DUSP16‐siRNA and ELK1‐siRNA (DUSP16‐shRNA, CAATCAGAAGGTGGTAGTTTA; ELK1‐shRNA, GAGAACAAGGTAATGGCCACATCAT; NC, TTCTCCGAACGTGTCACGTAA) obtained from Hanbio Biotechnology (Shanghai, China). In addition, the overexpression plasmids (LV‐DUSP16 and LV‐ELK1) and control vector were provided by Hanbio Biotechnology. The stable knockdown and overexpressed cell lines were selected by 8 μg/mL puromycin (Beyotime) for 48 h.

### 4.12 RT‐qPCR analyses

Initially, RNA was extracted from C17.2 cells using the kit from Beyotime Biotechnology. Subsequently, using the Tsingke Biotechnology kit, the first‐strand complementary DNA (cDNA) was generated through the process of reverse transcription. The mRNA expression was quantified using a Step One Real‐Time PCR System (Life Tech). Then the amplified aliquots of first‐stranded cDNA were obtained by applying gene‐specific primers and Power SYBR Green PCR Master Mix (Vazyme). Each PCR reaction contained 2 mg of cDNA, Universal Master Mix (Vazyme), and 10 mM of gene‐specific forward and reverse primers, with a final reaction volume of 20 μL. The mRNA expressions from different samples were exported from the data analysis software of the Real‐Time PCR system. The used primer sequences are provided in Table S3.

### 4.13 Computational analysis of ELK1 binding site in DUSP16 promoter

The promoter regions of DUSP16 were obtained from NCBI database (http://www.ncbi.nlm.nih.gov). The ELK1 binding sites of the DUSP16 promoter were predicted by the software PROMO, which can be accessed at https://alggen.lsi.upc.es/cgi‐bin/promo_v3/promo/promoinit.cgi?dirDB=TF_8.3.

### 4.14 ChIP‐qPCR assay

Briefly, cells were inoculated in 150 cm petri dishes with 20 mL of preheated complete medium. The culture dishes were then transferred to the incubator, and the medium was changed every 48 h until the cells reached approximately 90% confluency. To initiate crosslinking, 37% formaldehyde solution was added to each petri dish and stand at room temperature for 10 min. Then a 10x glycine solution was added to the culture media for 5 min. After washing, cells were collected and suspended in 1xChIP lysis buffer on ice for 15 min. Subsequently, the samples were resuspended in nuclear lysis/ultrasonic buffer for 10 min. Sonication was performed to fragment chromatin to an average length of 0.2–0.5 kb. After DNA purification, when the samples final DNA concentration reached 180 ng/mL, ELK1 (Abcam), histone H3, and IgG antibodies were added to each immunoprecipitation (IP) sample and incubated at 4°C overnight. Next, we placed 30 μL of protein G magnetic beads into the samples and incubated them at 4°C for 2 h for IP. The immunocomplexes were subsequently rinsed with low‐ and high‐salt rinse buffers, respectively. Finally, the samples were eluted and incubated with NaCl and protease K at 65°C overnight to reverse the crosslinking. The DNA purification step was repeated for subsequent qPCR analysis. The primers used for ChIP‐qPCR analysis are listed in Table S3.

### 4.15 Luciferase reporter assay

In ChIP experiments, HEK293T cells underwent transfection with a pGL3‐Basic Firefly luciferase reporter (Genomeditech Biotechnology) containing the DUSP16 promoter and a renilla luciferase vector. Additionally, the transient transfection expression vector pCDNA3.1, including the CDS sequence of ELK1 or the control sequence, also transfected into the HEK293T cells. Following a 72h incubation, the cells were lysed using a double luciferase reporter gene test kit (Beyotime Biotechnology), and the luciferase activity was measured.

### 4.16 Statistical analysis

Statistical analysis was conducted using GraphPad software and SPSS 21. The impact of SNPs on AD risk was assessed using R (4.2.2). A chi‐square test was used when comparing the distribution difference of DUSP16 SNPs between the MCI‐AD group and the NMCI‐AD group. The difference between the two groups was compared using a two‐tailed and unpaired *t*‐test. One‐ or two‐way analyses of variance (ANOVAs) were performed for data with multiple groups or for multifactorial analyses. Unless otherwise specified, the data are presented as mean ± SEM. Values of *p* < 0.05 were regarded as significant.

## AUTHOR CONTRIBUTIONS

Huimin Zhao, Xiaoquan Liu, and Haochen Liu conceived the concept and designed the experiments. Huimin Zhao analyzed data and wrote the manuscript. Huimin Zhao, Yao Mu, Anqi Liang, Jie Wei, Sixian Lai, Xin Li, and Peipei Chen performed all in vitro and in vivo experiments. Huimin Zhao, Xiaoquan Liu, and Haochen Liu designed the ADNI study. Hao Li, Hua He, Xiaoquan Liu, and Haochen Liu provided critical revision of the manuscript.

## FUNDING INFORMATION

This study was supported by the National Natural Science Foundation of China (No. 81903703) and the Inheritance Studio Construction Project of the National Famous Old Chinese Medicine Experts of Dr. Gu Zhaojun (Letter of the Department of Personnel and Education, the National Administration of Traditional Chinese Medicine, No.[2022]75). Data collection and sharing for this project were funded by the ADNI (NIH grant no. U01 AG024904 and Department of Defense award no.W81XWH‐12‐2‐0012). The investigators within the ADNI contributed to the design and implementation of ADNI or provided data but did not participate in the analysis or writing of this study. A complete listing of ADNI investigators can be found at http://adni.loni.usc.edu/wp‐content/uploads/how_to_apply/ADNI_Acknowledgement_List.pdf. ADNI is funded by the National Institute on Aging, the National Institute of Biomedical Imaging and Bioengineering, and through generous contributions from the following: AbbVie, Alzheimer's Association, Alzheimer's Drug Discovery Foundation, Araclon Biotech, BioClinica Inc., Biogen, Bristol‐Myers Squibb Company, CereSpir Inc., Cogstate, Eisai Inc., Elan Pharmaceuticals Inc., Eli Lilly and Company, EuroImmun, F. Hoffmann–La Roche Ltd. and its affiliated company Genentech Inc., Fujirebio, GE Healthcare, IXICO Ltd., Janssen Alzheimer Immunotherapy Research and Development LLC., Johnson & Johnson Pharmaceutical Research & Development LLC., Lumosity, Lundbeck, Merck & Co. Inc., Meso Scale Diagnostics LLC., NeuroRx Research, Neurotrack Technologies, Novartis Pharmaceuticals Corporation, Pfizer Inc., Piramal Imaging, Servier, Takeda Pharmaceutical Company, and Transition Therapeutics. The Canadian Institutes of Health Research is providing funds to support ADNI clinical sites in Canada. Private sector contributions are facilitated by the Foundation for the NIH (www.fnih.org). The grantee organization is the Northern California Institute for Research and Education, and the study is coordinated by the Alzheimer's Therapeutic Research Institute at the University of Southern California. ADNI data are disseminated by the Laboratory for NeuroImaging at the University of Southern California.

## CONFLICT OF INTEREST STATEMENT

The authors declare that they have no competing interests.

## Supporting information


Appendix S1.

## Data Availability

All data are available in the main text or the supplementary materials. Upon request, raw data are available from the corresponding author.
