# Supplementary material for: Suppressing DUSP16 overexpression induced by ELK1 promotes neural progenitor cell differentiation in mouse models of Alzheimer's disease
Source: Aging Cell. 2024 Oct 21;24(2):e14372. doi: 10.1111/acel.14372 (PMC11822628; doi:10.1111/acel.14372)
Supplement: Supplementary file 1 — Appendix S1. [file ACEL-24-e14372-s001.docx]

**Suppressing DUSP16 overexpression induced by ELK1 Promotes Neural Progenitor Cell Neural Differentiation in Mouse Models of Alzheimer's disease**

**Authors:** Huimin Zhao^1^, Yao Mu^1^, Anqi Liang^1^, Jie Wei^1^, Sixian Lai^1^, Xin Li^1^, Peipei Chen^1^, Hao Li^2^, Hua He^1,*^, Xiaoquan Liu^1,*^, Haochen Liu^1,*^

**Affiliations:**

^*^ *Corresponds author*

Address:

*^1^ Center of Drug Metabolism and Pharmacokinetics, China Pharmaceutical University, Nanjing, 210009, China*

*^2^Acupuncture and Moxibustion Department, Jiangsu Provincial Second Chinese Medicine Hospital/The Second Affiliated Hospital of Nanjing University of Chinese Medicine, Nanjing, 210017, China.*

Tel.: + 86-25-83271260

*E-Mail address: 3120010076@stu.cpu.edu.cn (HMZ); mynplmynpl@163.com (LM); 1434167014@qq.com(AQL); 1976614498@qq.com (JW); 2638416720@qq.com (SXL);1738475256@qq.com(XL); 1191065374@qq.com (PPC); lihao820719@163.com(HL);huahe_cpupk@cpu.edu.cn (HH); lxq@cpu.edu.cn(XQL), haochenliu@cpu.edu.cn(HCL) (HH, XQL, HCL Corresponds author).*

**List of Supplementary Materials**

Materials and Methods

Fig S1. DUSP16 regulates neural differentiation through the JNK-SOX2 pathway in SAMP8 and 3xTg mice.

Fig S2. Silencing DUSP16 rescues the impaired neural differentiation of NPCs in SAMP8 mice.

Fig S3. Sample immunofluorescence images of BrdU^+^/DCX^+^ cells in the dentate gyrus of C57BL/6 and 3xTg mice.

Fig S4. Silencing DUSP16 promotes synaptic density and a correlation analysis between elevated DUSP16 expression and the neural differentiation of NPCs.

Fig S5. Silencing DUSP16 increases the number of glutamatergic and GABAergic neurons and has no effect on the NPC pool in AD model mice.

Fig S6. Inhibition of DUSP16 expression alleviates deficient basal hippocampal transmission and LTP in SAMP8 mice.

Fig S7. Inhibition of DUSP16 expression improves memory impairments in SAMP8 mice.

Fig S8. DUSP16 regulates neural differentiation through the JNK-SOX2 pathway in C17.2 cells.

Fig S9. DUSP16 inhibition has no effect on Aβ deposition.

Fig S10. The alterations of ELK1 protein expression in SAMP8 mice and C17.2 cells.

Fig S11. The regulation of DUSP16 on NPCs neural differentiation under the AD state.

Tables S1. Participants in ADNI cohort.

Tables S2. The comparison of two clusters in the MCI-AD group and the NMCI-AD group.

Tables S3. The Sequences of primers used for Real-time PCR reactions in mouse species.

**Supplementary Materials**

**
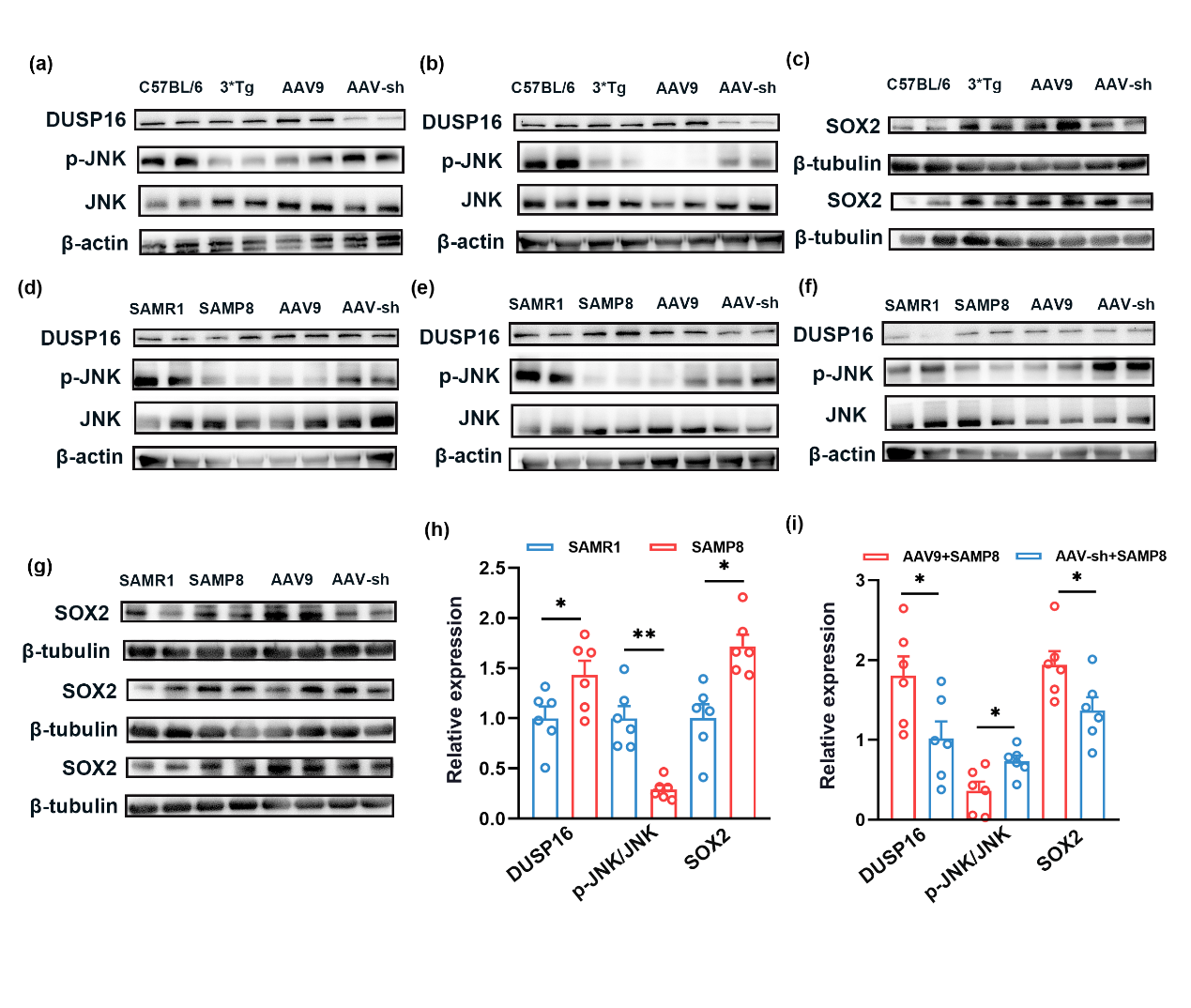
**

**Supplementary figure 1.** **DUSP16 regulates neural differentiation through the JNK-SOX2 pathway in SAMP8 and 3xTg mice.** (**a** to **b**) Western blot images of DUSP16, p-JNK/JNK ratio and SOX2 in the control group (C57BL/6), the AD group (3xTg), the AAV9 group (3xTg) and the AAV-shRNA group (3xTg) (n=6). (**d** to **i**) Western blot images and quantitative analyses of DUSP16, p-JNK/JNK ratio and SOX2 in the control group (SAMR1), the AD group (SAMP8), the AAV9 group (SAMP8) and the AAV-shRNA group (SAMP8) (n=6). **p* < 0.05; ** *p* < 0.01; *** *p* < 0.001, Student’s t test was used for all data analyses, Data are presented as mean ± SEM.

**
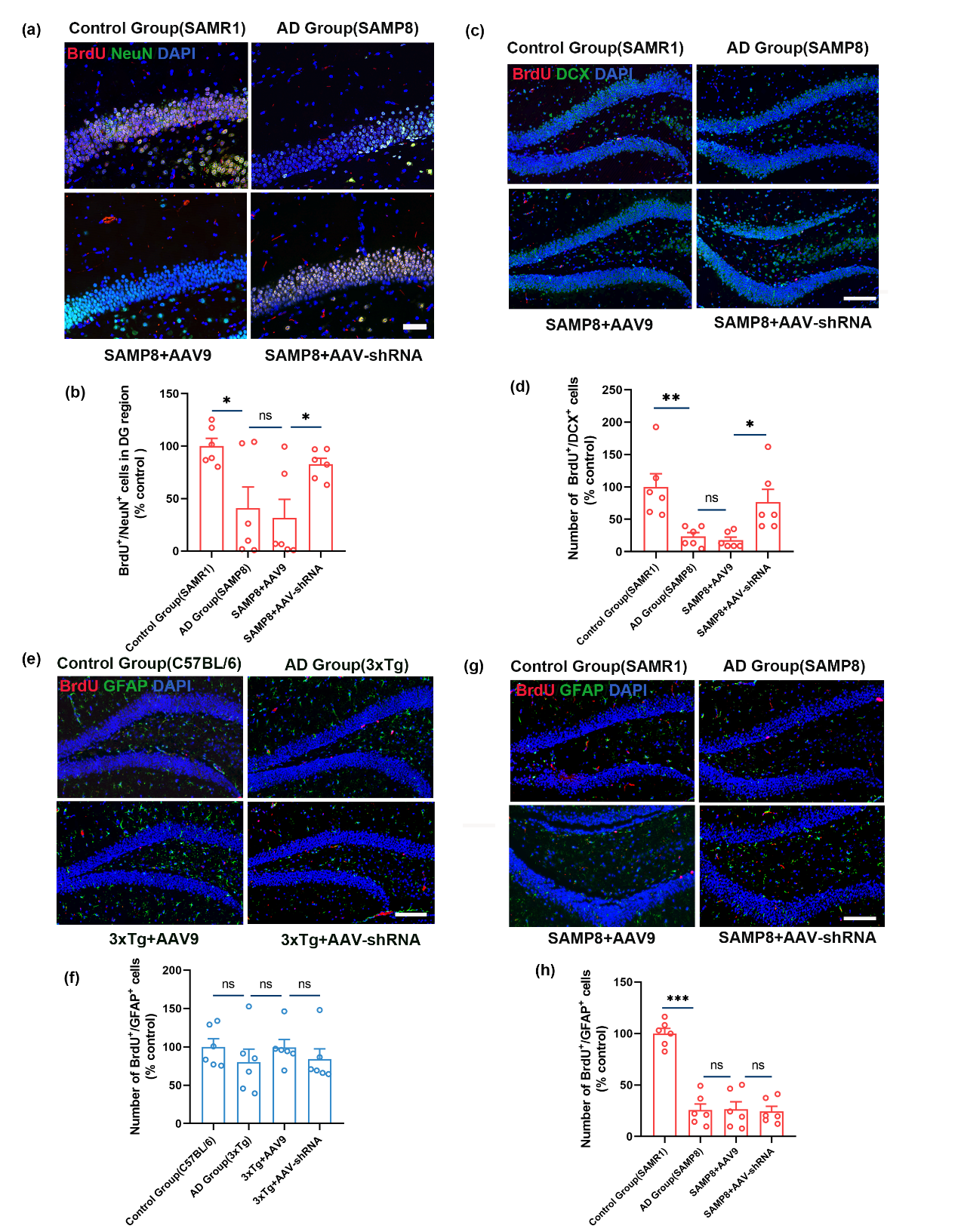
**

**Supplementary figure 2.** **Silencing DUSP16 rescues the impaired neural differentiation of NPCs in SAMP8 mice.** (**a** and **b**) Sample immunofluorescence images of BrdU^+^/NeuN^+^ cells in the SAMR1 and SAMP8 mice dentate gyrus, and quantitative comparison of the percentage of BrdU^+^/NeuN^+^ cells in the DG region of SAMR1 and SAMP8 mice (n = 6 per group). The SAMP8 mice were treated with AAV9 and AAV-DUSP16, or without treatment. Blue, DAPI; green, NeuN; red, BrdU. Scale bars, 50 μm. (**c** and **d**) Sample immunofluorescence images of BrdU^+^/DCX^+^ cells in the dentate gyrus of SAMR1 and SAMP8 mice, and quantitative comparison of the percentage of BrdU^+^/DCX^+^ cells in the DG region of SAMR1 and SAMP8 mice (n = 6 per group). The SAMP8 mice were treated with AAV9 and AAV-DUSP16, or without treatment. Blue, DAPI; green, DCX; red, BrdU. Scale bars, 100 μm. (**e** and **f**) Immunofluorescence images (e) and quantitative analysis (f) showing that there was no difference in the number of new-born astrocytes among the four groups mice (3xTg). Blue, DAPI; green, GFAP; red, BrdU. Scale bars, 100 μm. (**g** and **h**) Immunofluorescence images (g) and quantitative analysis (h) showing that there was no difference in the number of new-born astrocytes among the AD group (SAMP8), the SAMP8+AAV9 group and SAMP8+AAV-shRNA group. Compared to the SAMR1 group, the number of new-born astrocytes significantly reduced in the remaining three groups. Blue, DAPI; green, GFAP; red, BrdU. Scale bars, 100 μm. * *p* < 0.05; ** *p* < 0.01; *** *p* < 0.001, Student’s t test was used for all data analyses, Data are presented as mean ± SEM.


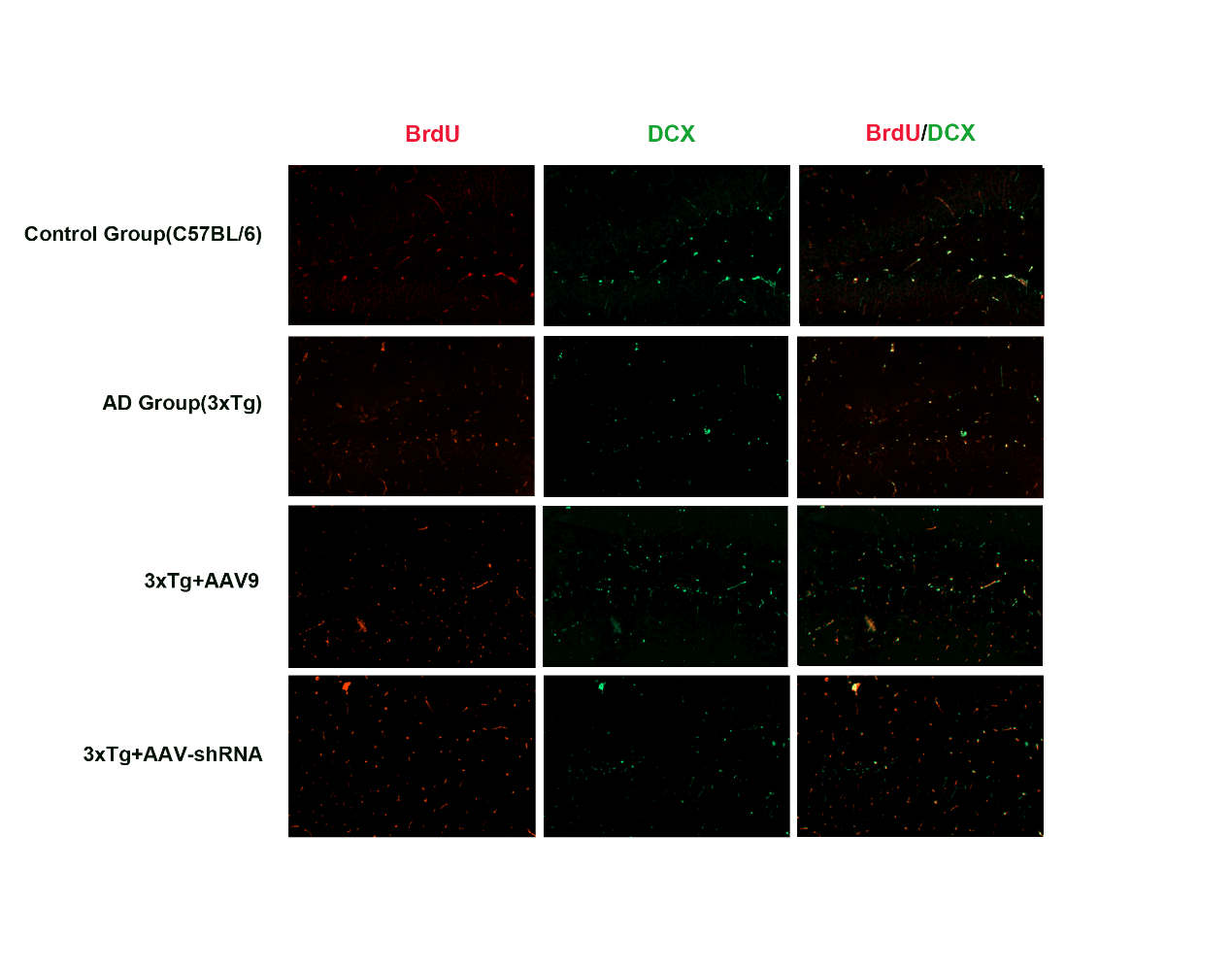


**Supplementary figure 3.** **Sample immunofluorescence images of BrdU^+^/DCX^+^ cells in the dentate gyrus of C57BL/6 and 3xTg mice.** Sample immunofluorescence images of BrdU^+^, DCX^+^ and BrdU^+^/DCX^+^ cells in the dentate gyrus of C57BL/6 and 3xTg mice (n = 6 per group). The 3xTg mice were treated with AAV9 and AAV-DUSP16, or without treatment. Green, DCX; red, BrdU. Scale bars, 100 μm.

**
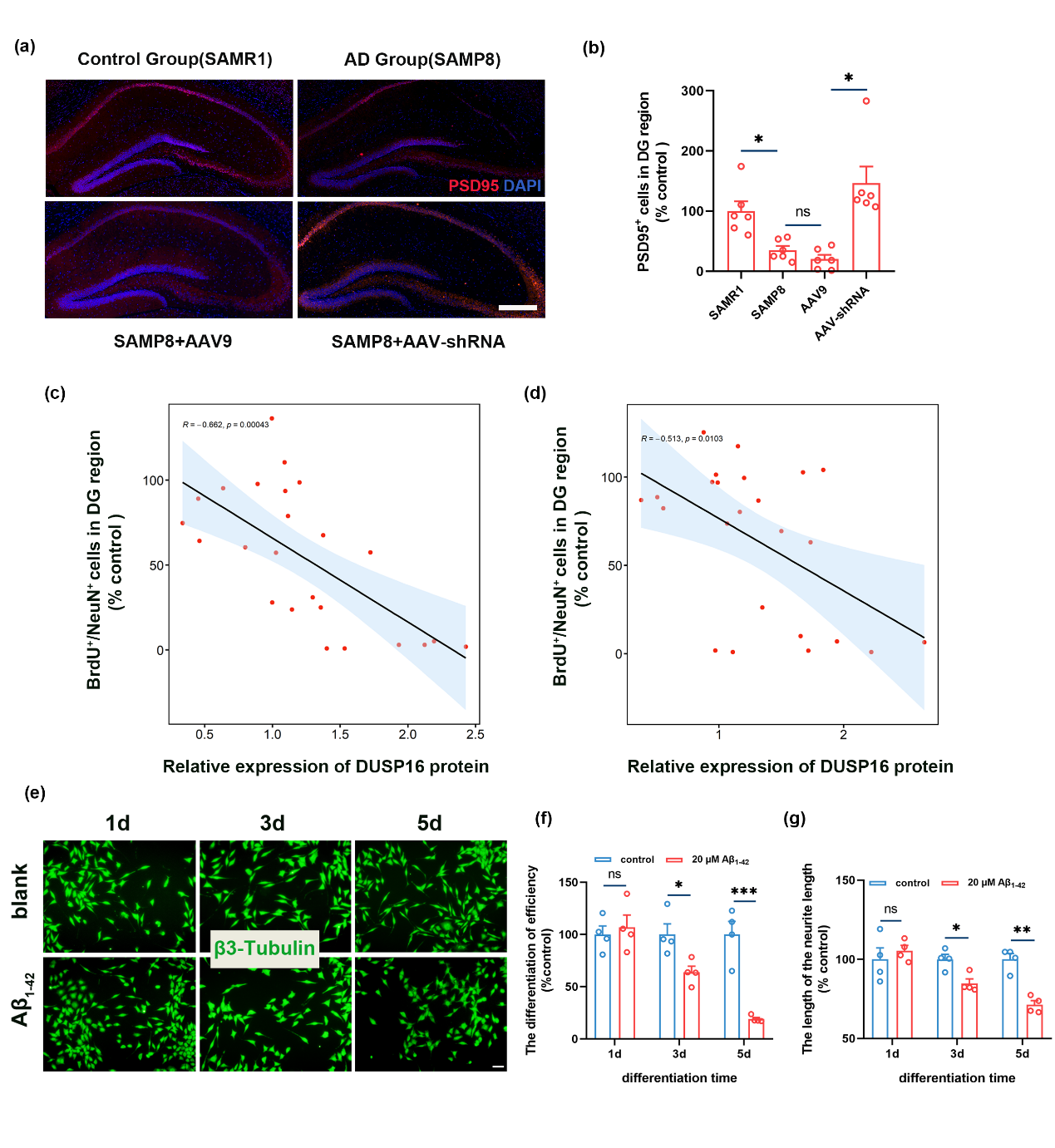
**

**Supplementary figure 4.** **Silencing DUSP16 promotes synaptic density and a correlation analysis between elevated DUSP16 expression and the neural differentiation of NPCs.** (**a** and **b**) Sample immunofluorescence images of PSD95^+^ cells in the hippocampus of SAMR1 and SAMP8 mice (n = 6 per group), followed by quantitative analysis of PSD95^+^ cells (b). The SAMP8 mice were treated with AAV9 and AAV-DUSP16, or without treatment. Blue, DAPI; red, PSD95. Scale bars, 200 μm. (**c**) Pearson's correlation analysis was performed between DUSP16 expression and the number of BrdU^+^/NeuN^+^ positive cells in the hippocampus of 3xTg mice (n = 6 per group). (**d**) Pearson's correlation analysis was performed between DUSP16 expression and the number of BrdU^+^/NeuN^+^ positive cells in the hippocampus of SAMP8 mice (n = 6 per group). (**e**) Representative images showing that C17.2 cells incorporated β3-Tubulin (green) and DAPI (blue) under differentiated times (1d, 3d, 5d), with or without Aβ_1-42_ treatment. Scale bar, 100 mm. (**f** and **g**) Quantitative analysis showing that Aβ_1-42_ treatment decreased the neural differentiation rate and neurite length of C17.2 cells (n = 4). * *p* < 0.05; ** *p* < 0.01; *** *p* < 0.001, Student’s t test was used in (b), (f) and (g), Pearson correlation analysis was used in (c) and (d). Data are presented as mean ± SEM.

**
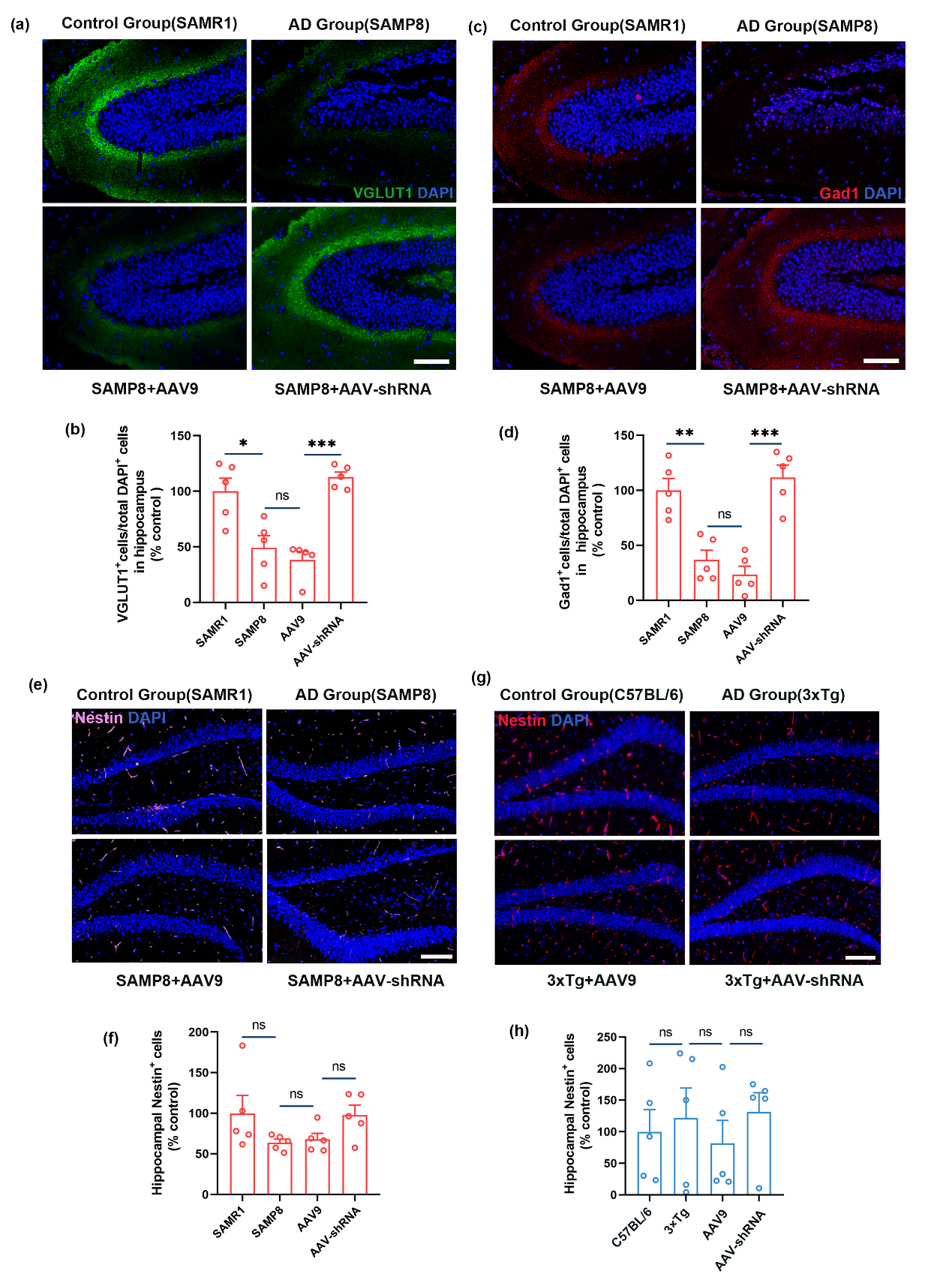
**

**Supplementary figure 5.** **Silencing DUSP16 increases the number of** **glutamatergic and GABAergic neurons and has no effect on the NPC pool in AD model mice.** (**a** and **b**) Sample immunofluorescence images of VGLUT1^+^ cells in the hippocampus of SAMR1 and SAMP8 mice (n = 5 per group), followed by quantitative analysis of VGLUT1^+^ cells (B). The SAMP8 mice were treated with AAV9 and AAV-DUSP16, or without treatment. Blue, DAPI; green, VGLUT1. Scale bars, 50 μm. (**c** and **d**) Sample immunofluorescence images of Gad1^+^ cells in the hippocampus of SAMR1 and SAMP8 mice (n = 5 per group), followed by quantitative analysis of Gad1^+^ cells (d). (**e** and **f**) Sample immunofluorescence images of Nestin^+^ cells in the hippocampus of SAMR1 and SAMP8 mice (n = 5 per group), followed by quantitative analysis of Nestin^+^ cells (f). The SAMP8 mice were treated with AAV9 and AAV-DUSP16, or without treatment. Blue, DAPI; pink, Nestin. Scale bars, 100 μm. (**g** and **h**) Sample immunofluorescence images of Nestin^+^ cells in the hippocampus of C57BL/6 and 3xTg mice (n = 5 per group), followed by quantitative analysis of Nestin^+^ cells (H). The 3xTg mice were treated with AAV9 and AAV-DUSP16, or without treatment. Blue, DAPI; red, Nestin. Scale bars, 100 μm. *P < 0.05, Student’s t test was used for all data analyses, Data are presented as mean ± SEM.

**
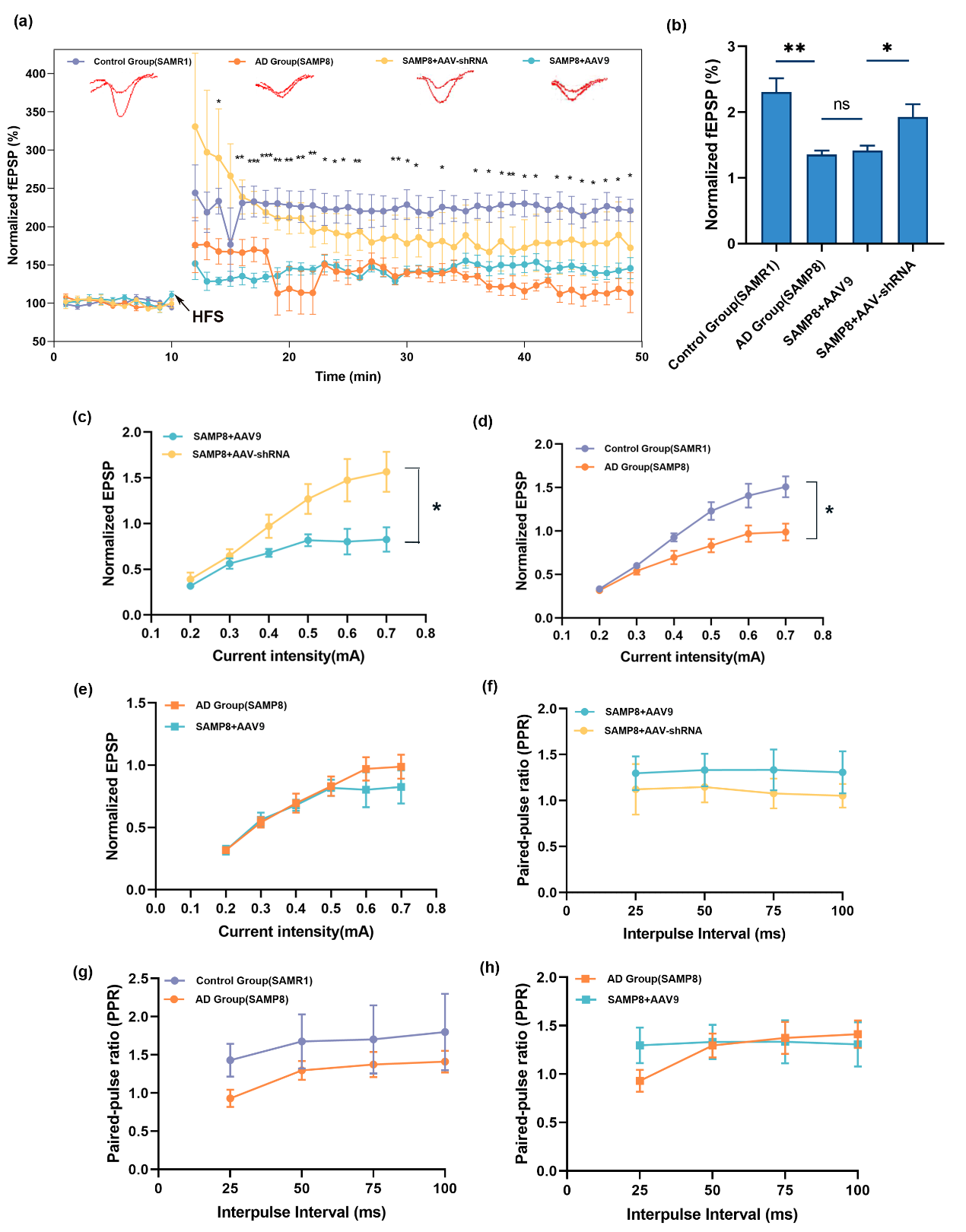
**

**Supplementary figure 6.** **Inhibition of DUSP16 expression alleviates deficient basal hippocampal transmission and LTP in SAMP8 mice.** (**a**) Normalized fEPSP amplitude to field stimulation before and after HFS in SAMR1 and SAMP8 mice treated with AAV-DUSP16 or AAV-9, or without treatment. The slope of the initial component of the fEPSP was normalized to baseline, and a baseline for 10 mins was obtained before HFS. (**b**) Mean value of potentiation between 12 and 50 min in four groups mice after HFS. (**c** to **e**) EPSPs at various intensities were observed in the input–output curve in SAMR1 and SAMP8 mice treated with AAV-DUSP16 or AAV-9, or without treatment. (**f** to **h**) PPR with different inter-stimulus intervals in SAMR1 and SAMP8 mice treated with AAV-DUSP16 or AAV-9, or without treatment. n = 5 mice per group, * *p* < 0.05; ** *p* < 0.01; *** *p* < 0.001. one-way ANOVA was used in (a); Student’s t test was used in (b); two-way ANOVA was used in (c) to (h). Data are presented as mean ± SEM.


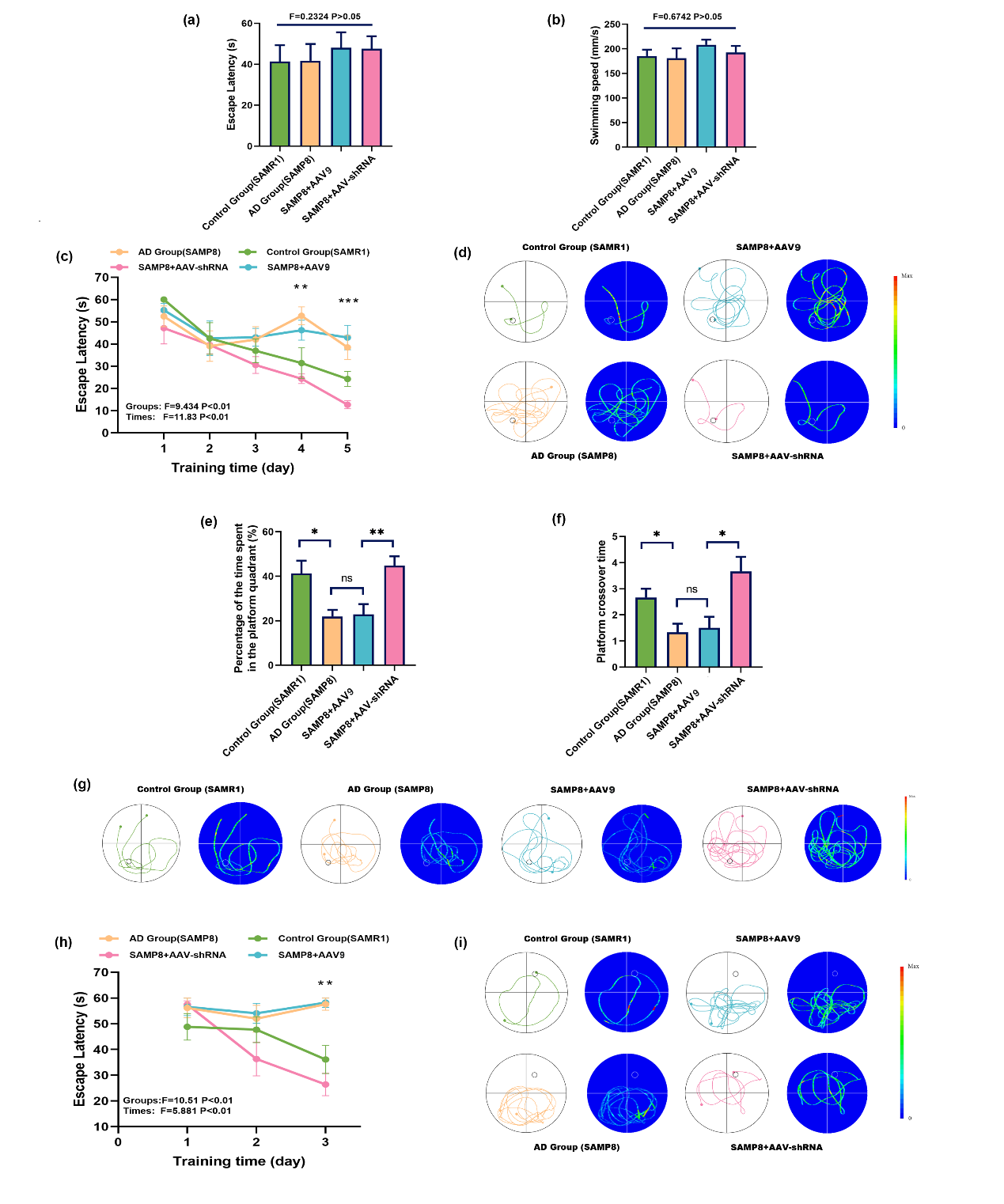


**Supplementary figure 7.** **Inhibition of DUSP16 expression improves memory impairments in SAMP8 mice.** (**a** to **b**) There were no difference among the four groups in the escape latency and swimming speed in the visible platform trail. (**c**) Silencing DUSP16 resulted in the decreased escape latency in SAMP8 mice in the hidden platform trail. (**d**) The heat maps of pooled animals revealed the results of the hidden platform trail. (**e** to **f**) Silencing DUSP16 resulted in the reduced platform crossover times and percentage of time spent in the platform quadrant in SAMP8 mice in the probe trail. (**g**) The heat maps of pooled animals revealed the results of the probe trail. (**h**) Silencing DUSP16 resulted in the decreased escape latency in SAMP8 mice in the reference trail. (**i**) The heat maps of pooled animals revealed the results of the reference trail. n = 6 mice per group, * *p* < 0.05; ** *p* < 0.01; *** *p* < 0.001. Student’s t test was used in (e) to (f); One-way ANOVA was used in (a), (b), (c) and (h). Two-way ANOVA was used in (c) and (h). Data are presented as mean ± SEM.


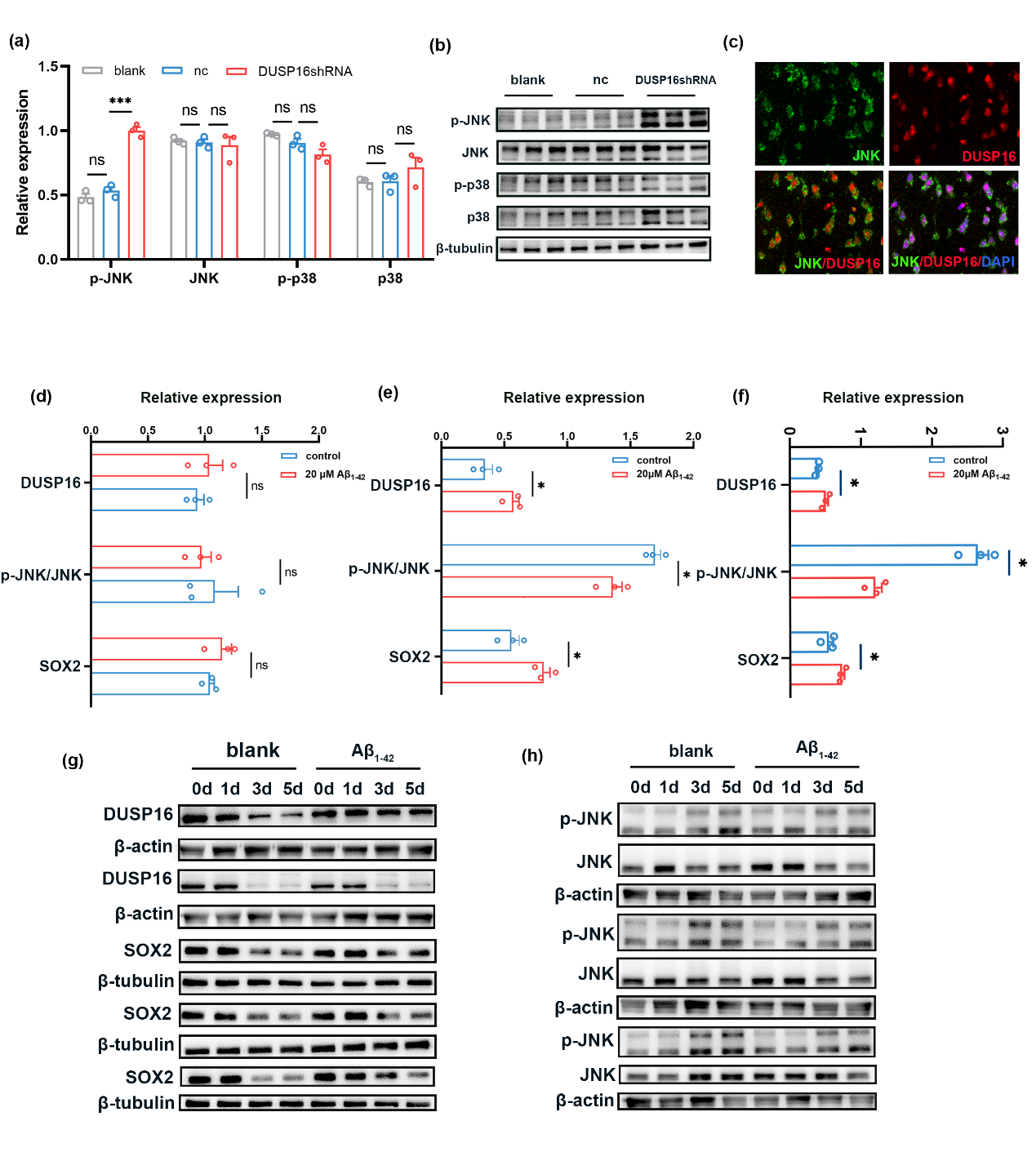


**Supplementary figure 8.** **DUSP16 regulates neural differentiation through the JNK-SOX2 pathway in C17.2 cells.** (**a** to **b**) Western blot analyses of p-JNK, p-JNK/JNK ratio, p-p38, and p38 in blank, NC and DUSP16-downregulated C17.2 cells (n = 3). (**c**) Sample confocal images of JNK^+^DUSP16^+^ cells in the hippocampus of SAMP8 mice, Scale bars, 50 μm. (**d** to **h**) Western blot analyses of DUSP16, p-JNK/JNK ratio and SOX2 in C17.2 cells at different differentiation time (1d (d), 3d (e), 5d(f)), with or without Aβ_1-42_ treatment (n = 3). * *p* < 0.05; ** *p* < 0.01; *** *p* < 0.001, Student’s t test was used for all data analyses, Data are presented as mean ± SEM.


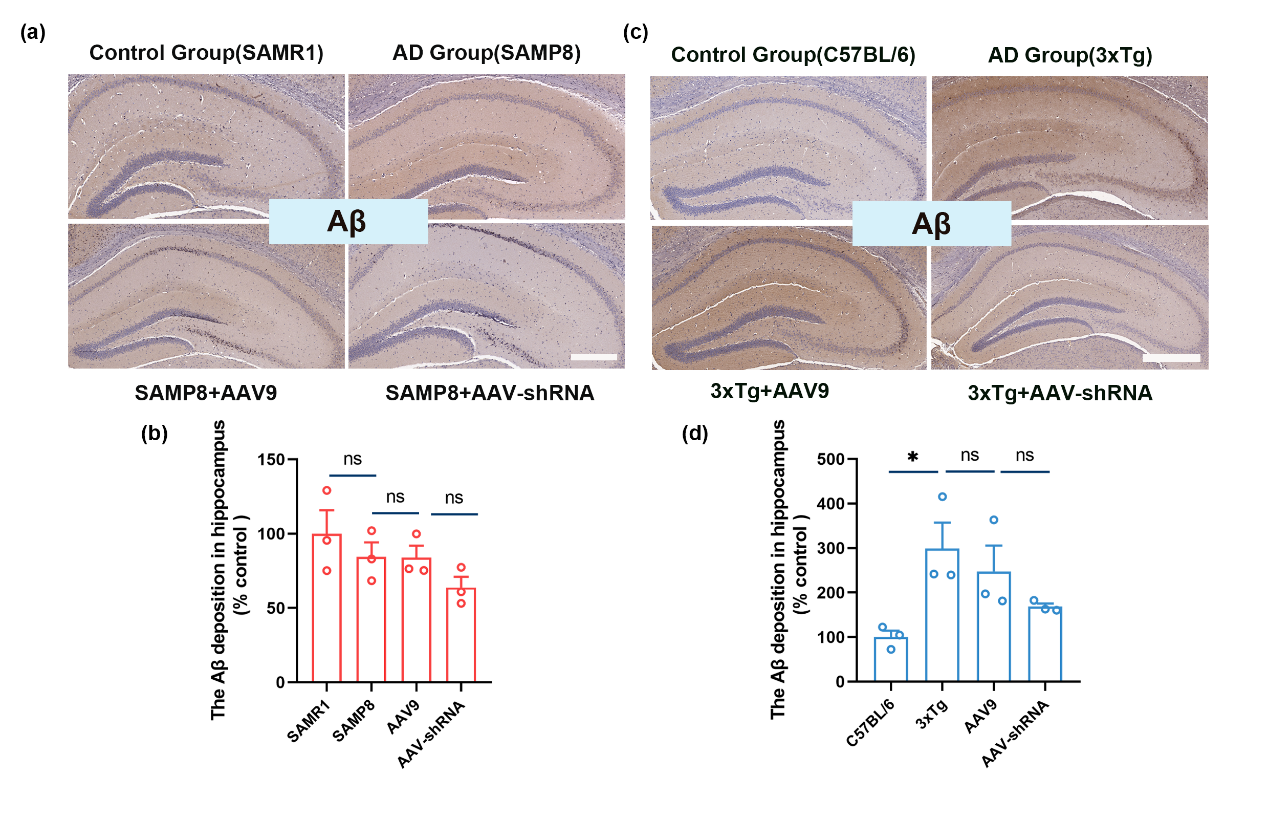


**Supplementary figure 9. DUSP16 inhibition has no effect on Aβ deposition.** (**a** and **b**) Representative immumohistochemical staining images of Aβ in the control group (C57BL/6), the AD group (3xTg), the AAV9 group (3xTg) and the AAV-shRNA group (3xTg), quantitative analysis in (b) (n=3). Scale bars, 200 μm. (**c** and **d**) Representative immumohistochemical staining images of Aβ in the control group (SAMR1), the AD group (SAMP8), the AAV9 group (SAMP8) and the AAV-shRNA group (SAMP8), quantitative analysis in (d) (n=3). Scale bars, 200 μm. * *p* < 0.05, Student’s t test was used for all data analyses, Data are presented as mean ± SEM.


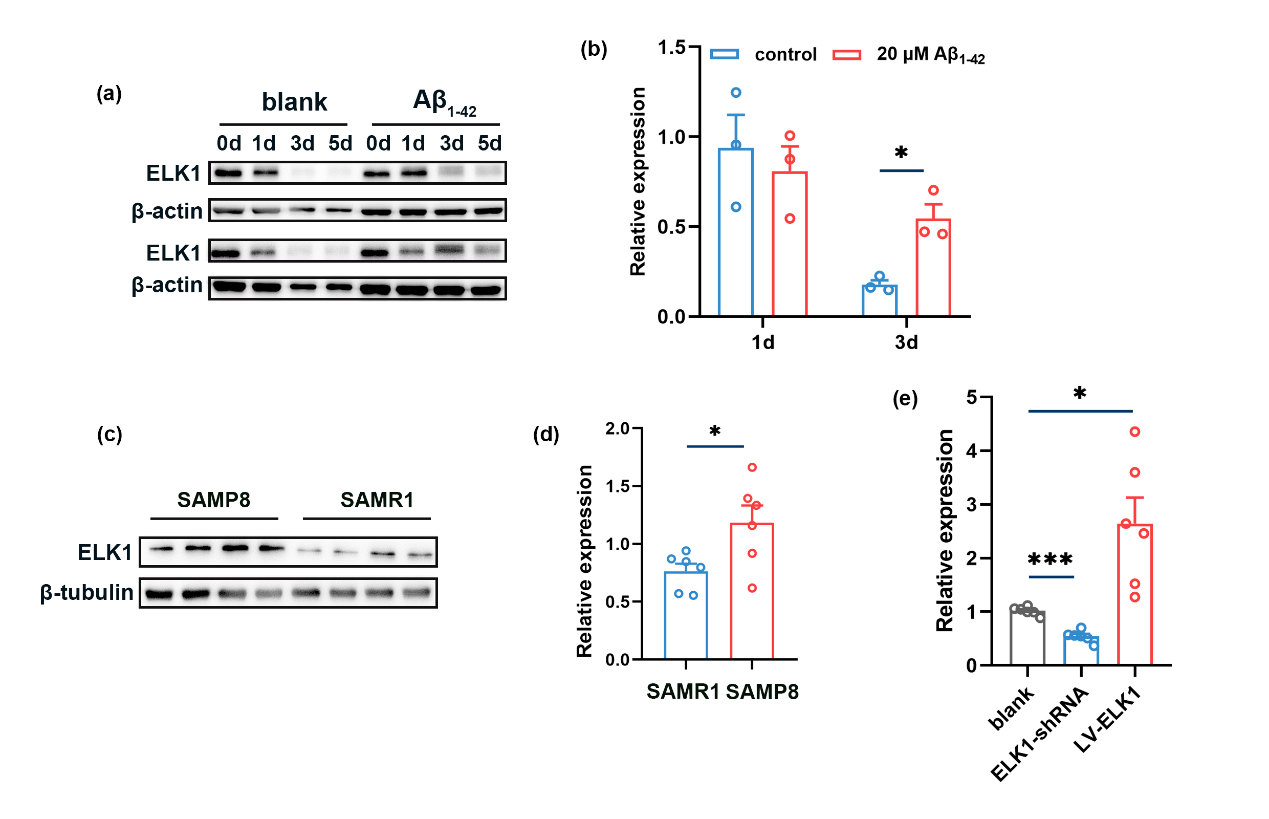


**Supplementary figure 10.** **The alterations of ELK1 protein expression in SAMP8 mice and C17.2 cells.** (**a** and **b**) Western blot analyses of ELK1 in C17.2 cells at different differentiation time (1d,3d), with or without Aβ_1-42_ treatment (n=3). (**c** and **d**) Western blot analyses of ELK1 in SAMP8 and SAMR1 mice (n=6). (**e**) Quantification analyses of ELK1 mRNA in ELK1-shRNA and LV-ELK1 C17.2 cells (n= 3). * *p* < 0.05, Student’s t test was used for all data analyses, Data are presented as mean ± SEM.


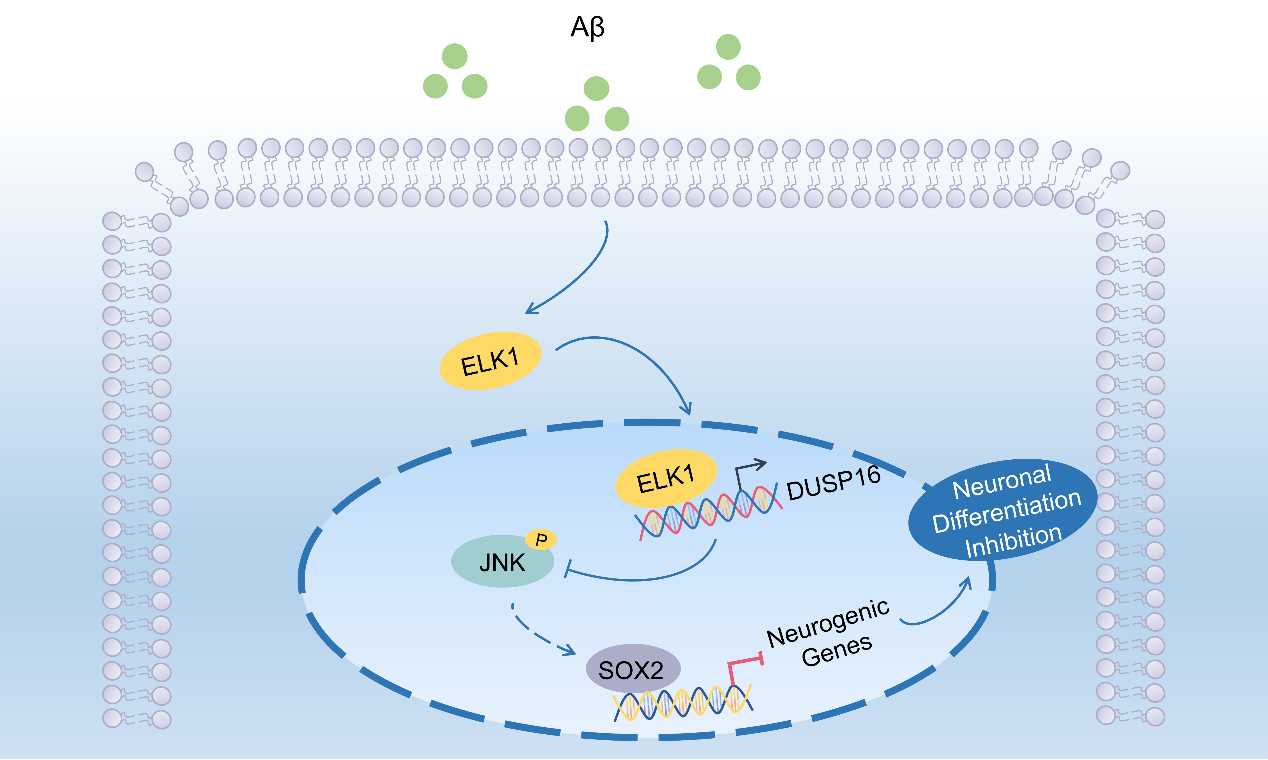


**Supplementary figure 11. The regulation of DUSP16 on NPCs neural differentiation under the AD state.** Schematic indicated that increased expression of DUSP16 leads to JNK deactivation and subsequent induction of SOX2 expression, ultimately leading to deficits in neural differentiation of NPCs under the AD state.

| Table S1 Participants in ADNI cohort | | | |  |
| --- | --- | --- | --- | --- |
|  | AD Group | MCI Group | Control Group | |
| Number | 48 | 480 | 281 | |
| Age | 75.51±9.23 | 72.29±7.46 | 74.62±5.57 | |
| Gender(male/female) | 18/30 | 280/200 | 136/145 | |
| ADAS-cog | 29.80±8.43 | 15.31±6.60 | 8.94±4.39 | |

Table S2 The comparison of two clusters in the MCI-AD group and the NMCI-AD group

| Clusters | The number of subjects in MCI-AD | The number of subjects in NMCI-AD | Chi-square test for p-values |
| --- | --- | --- | --- |
| harboring 0 SNP | 40 | 75 | 0.033 |
| harboring 7 SNPs | 8 | 40 |  |

|  |
| --- |
|  |

Table S3 The Sequences of primers used for Real-time PCR reactions in mouse species

| Gene | The Sequences of primers |
| --- | --- |
| ELK1 | Forward: AGGGATCAAACGGTCACCTT  Reverse: AAAGACGTGTGCCTCTACCA |
| DUSP16 (ChIP-qPCR) | Forward: TAATTCTTGGCTGCGTTTGCC  Reverse: AAGGAAAGCAAGGCGATCTGA |
| DUSP16 (PCR) | Forward: ACCGCATGGACCCTTTACTT  Reverse: GACCCTACTACACGAGTGCG |
